# Transcriptomic Signatures of Postnatal and Adult Intrinsically Photosensitive Ganglion Cells

**DOI:** 10.1523/ENEURO.0022-19.2019

**Published:** 2019-08-23

**Authors:** Daniel J. Berg, Katherine Kartheiser, Megan Leyrer, Alexandra Saali, David M. Berson

**Affiliations:** 1 Molecular Biology, Cellular Biology, and Biochemistry Program, Brown University, Providence, Rhode Island 02912; 2 Department of Neuroscience, Brown University, Providence, Rhode Island 02912

**Keywords:** cell type, circadian, intrinsically photosensitive, retinal ganglion, RNA-seq, vision

## Abstract

Intrinsically photosensitive retinal ganglion cells (ipRGCs) are rare mammalian photoreceptors essential for non-image-forming vision functions, such as circadian photoentrainment and the pupillary light reflex. They comprise multiple subtypes distinguishable by morphology, physiology, projections, and levels of expression of melanopsin (Opn4), their photopigment. The molecular programs that distinguish ipRGCs from other ganglion cells and ipRGC subtypes from one another remain elusive. Here, we present comprehensive gene expression profiles of early postnatal and adult mouse ipRGCs purified from two lines of reporter mice that mark different sets of ipRGC subtypes. We find dozens of novel genes highly enriched in ipRGCs. We reveal that *Rasgrp1* and *Tbx20* are selectively expressed in subsets of ipRGCs, though these molecularly defined groups imperfectly match established ipRGC subtypes. We demonstrate that the ipRGCs regulating circadian photoentrainment are diverse at the molecular level. Our findings reveal unexpected complexity in gene expression patterns across mammalian ipRGC subtypes.

## Significance Statement

A comprehensive transcriptomic analysis has identified dozens of genes differentially expressed in intrinsically photosensitive retinal ganglion cells, including some linked to signaling, gene regulation, and melanopsin phototransduction.

## Introduction

Many unique attributes distinguish intrinsically photosensitive retinal ganglion cells (ipRGCs) from conventional RGCs. Only ipRGCs express the blue-light-sensitive photopigment melanopsin (OPN4), which renders them autonomously light-sensitive. They violate the usual stratification rule in which ON-type RGCs deploy their dendrites only in the inner (proximal) half of the inner plexiform layer; their inputs from ON bipolar cells are atypical (Dumitrescu et al., 2009; Hoshi et al., 2009; Kim et al., 2010). Whereas most RGCs direct their entire output through the optic nerve, some ipRGCs modulate intra-retinal processing, through amacrine cells (Zhang et al., 2008; Xue et al., 2011; Reifler et al., 2015; Sabbah et al., 2017) and spontaneous retinal waves during the early postnatal period (Renna et al., 2011). Functionally, ipRGCs are unique among RGCs in their ability to encode overall light intensity for extended periods (Wong, 2012). This tonic luminance signal is transmitted to specific brain targets for a variety of functions including photoentrainment of circadian rhythms and light-evoked pupillary constriction. Additionally, ipRGCs appear more resistant than RGCs overall to various insults, including optic nerve injury, glutamate-induced excitotoxicity, and glaucoma (Cui et al., 2015).

**Figure 1.**
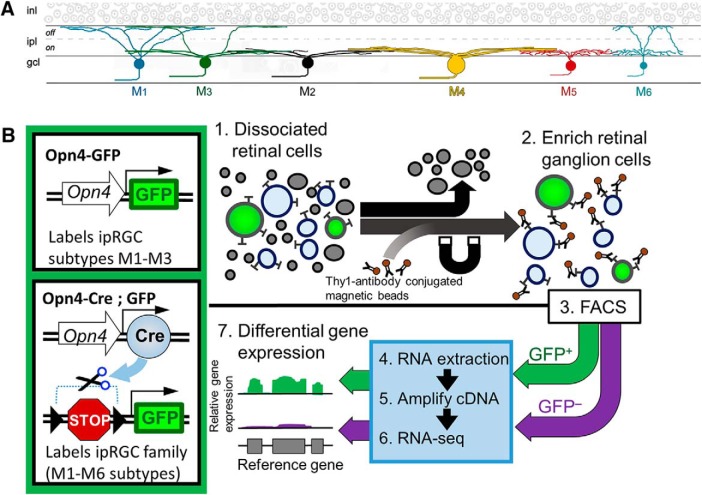
Experimental design of gene expression profiling from purified ipRGCs and comparison with generic RGCs. ***A***, Current model of ipRGC family members integrating molecular, physiology, brain circuitry, and morphology (see text for details). ***B***, Two transgenic reporters were used for gene expression profiling of ipRGCs. The BAC transgenic *Opn4-GFP* labels M1–M3 ipRGCs, whereas the *Opn4-Cre* crossed with a cre-dependent GFP reporter labels M1–M6 ipRGCs. Within the schematic of the gene expression profiling procedure: (1) Isolation of cell populations from enzymatically dissociated retinas. (2) The surface protein Thy-1 is enriched in RGCs, this high affinity of Thy1-conjugated magnetic beads to RGCs was used to enrich the extracted cell populations with RGCs. (3) FACS was used to isolate GFP-positive cells (ipRGCs) from GFP-negative cells (cRGCs). These two populations were isolated in parallel to provide direct internal testing of ipRGCs versus cRGCs under the same treatments, conditions, and genetic backgrounds. (4) The RNA of these two main populations was subjected to mRNA extraction. (5) The RNA was converted to cDNA and amplified using Nugen Ovation RNA amplification system. (6) Illumina TruSeq sequencing libraries were prepared by ligating adapters to the cDNA. Single-end 50 bp sequencing was completed using the Illumina HiSeq system. (7) DEGs were determined using EdgeR bioinformatics pipeline. See Materials and Methods for details.

The ipRGCs consist of at least six anatomically distinct retinal subtypes, termed M1–M6 (Fig. 1*A*). These differ in their level of melanopsin expression, visual response properties, dendritic stratification, axonal projections, and contributions to light-modulated behavioral responses (Schmidt et al., 2011). The suprachiasmatic nucleus (SCN) receives projections primarily from M1 ipRGCs, but also a minor input from the M2 subtype (Viney et al., 2007; Baver et al., 2008). The ipRGCs project to distinct regions of the midbrain, with M1 and M2 ipRGC axons arriving to the shell and core of the olivary pretectal nucleus (OPN), respectively (Prichard et al., 2002; Baver et al., 2008; Güler et al., 2008). The OPN also receives significant input from the recently described M6 ipRGCs (Quattrochi et al., 2019). M2–M4 ipRGCs send projections to image-forming brain regions and mediate coarse pattern vision, whereas spectrally opponent M5 cells contribute to color vision (Ecker et al., 2010; Estevez et al., 2012; Sonoda et al., 2018; Stabio et al., 2018).

The distinctive structural and functional properties of ipRGCs must ultimately be traceable to different patterns of gene expression that have remained elusive. The melanopsin phototransduction cascade is a major defining feature of ipRGCs and the basic molecular framework has been identified (for review, see Hughes et al., 2012). However, the precise phototransduction mechanisms across the ipRGC subtypes have only recently become characterized (Jiang et al., 2018; Sonoda et al., 2018). M1 ipRGCs have been further subdivided based on their expression of the transcription factor Pou4f2 (Brn3b; Chen et al., 2011; Jain et al., 2012). Ablation of Brn3b-positive ipRGCs severely impairs the pupillary light reflex, but leaves circadian photoentrainment intact (Chen et al., 2011). Further, Brn3b-positive M1 ipRGCs provide inputs to diverse brain regions including the thalamus, midbrain, and hypothalamus (Li and Schmidt, 2018). Additionally, the transcription factor Tbr2 is selectively expressed in adult ipRGCs (Mao et al., 2014; Sweeney et al., 2014). Further molecular diversity is expected among ipRGCs, both within and between established ipRGC subtypes.

Previous attempts to develop a “molecular parts list” for ipRGCs through gene-expression profiling of adult ipRGCs have been limited by the extreme heterogeneity of retinal tissue and the fragility of mature retinal neurons (Lobo et al., 2006; Heiman et al., 2008; Sanes and Masland, 2015). Prior efforts using either anti-melanopsin immunopanning or fluorescence-activated cell sorting (FACS) of genetically-labeled fluorescent ipRGCs have been limited by low yield and inclusion of contaminating cell populations such as rods (Hartwick et al., 2007; Peirson et al., 2007; Siegert et al., 2012).

Here we conducted a thorough unbiased transcriptomic analysis of ipRGCs by purifying green fluorescent protein (GFP)-tagged ipRGCs through a combination of FACS and immuno-affinity and comparing with the transcriptional profile of GFP-negative RGCs. We did this in two different mouse lines, marking partially overlapping subsets of ipRGCs. The specificity and purity of these ipRGC samples is validated by their substantial enrichment for transcripts of genes known to be selectively expressed in ipRGCs and very low expression levels of genes linked to potentially contaminating cell types. We identified >75 new gene candidates expressed much more highly in adult ipRGCs than in other RGCs. We validate two of the new molecular markers at the protein level: Rasgrp1, which is a Ras guanine nucleotide exchange factor (GEF); and Tbx20, a T-box transcription factor. We relate these novel markers to established ipRGC subtypes and patterns of central projection.

## Materials and Methods

### Animals

All experiments were conducted in accordance with NIH guidelines under protocols approved by the Brown University Animal Care and Use Committee. Both male and female adult mice [postnatal day (P)30–P90] were used unless otherwise stated. *Opn4^cre/cre^* mice were crossed with floxed-stop reporter mice: “*Z/EG*” (*Jax#003920*); the offspring express GFP in *cre*-expressing cells (M1–M6), as described by Ecker et al., 2010. *Opn4-GFP(ND100Gsat)* is a BAC transgenic mouse generated by the GENSAT project at Rockefeller University. The Rasgrp1-KO (Rasgrp1^tm1Jstn^; Dower et al., 2000) was initially provided generously by Robert Barrington (University of Alabama) for initial testing. The *Cdh3-GFP* reporter is a BAC transgenic originally generated by the Gensat project (MMRRC, BK102Gsat/MMNC) useful for identifying M6 ipRGCs (Quattrochi et al., 2019). This mouse line was backcrossed with C57BL/6J background for >10 generations. *Cdh3-GFP* mice were 3 weeks old or younger unless otherwise stated.

### Retinal dissociation

Mice from either P5 (± 1day) or young adult (P30 ± 3 days) mice were euthanized by inhalation of CO_2_. Retinal tissue was dissected free into room temperature Hibernate-A medium (BrainBits). At least three biological replicates were produced for each dataset: three replicates for immature Gensat *Opn4-GFP* mice (P4–P6); six for adult Gensat *Opn4-GFP* mice; and four for *Opn4^Cre/+^; Z/EG* ^+/−^. We were able to regularly isolate >10,000 GFP+ ipRGCs from nine P5 transgenic reporter mice. In contrast, each adult replicate required 15–20 mice to obtain sufficient cells for the transcriptional analysis because of the relative fragility of adult RGCs.

Fresh retinas were enzymatically dissociated in a medium containing 10 ml of Hibernate-A minus Calcium (BrainBits), 20U/ml papain (Worthington), 0.25% GlutaMAX (Invitrogen), 1 mm l-cysteine, 0.004% DNase, and titrated to 7.4 pH with NaOH. The dissociation medium was activated for 30 min at 37°C before retinal immersion. Retinas were incubated in it for 45 min, with gentle shaking every 5–10 min, then centrifuged for 3 min at 200 rcf and washed with 1 ml of trituration medium containing Hibernate-A and 10% fetal calf serum. Retinas were gently triturated 10–15 times with a P1000 tip and an additional 4 ml of trituration buffer was added to each tube. The retina cell suspension was centrifuged for 11 min at 1000 rcf. The pellet was washed and resuspended with 1ml of HABG buffer containing Hibernate-A, 0.25% BSA, 1% B27 (Invitrogen), and 0.25% GlutaMAX (Invitrogen).

### RGC pre-enrichment

As a first step to purifying ipRGCs from dissociated retinal cells, we selected for ganglion cells by immuno-affinity for the cell-surface protein Thy-1 (Barres et al., 1988; Cahoy et al., 2008). We adapted classic immunopanning procedures to a magnetic-activated cell-sorting approach (Miltenyi Biotec). We incubated 1 ml of dissociated retinal cell suspension with 100 µl Thy1(CD90.2)-conjugated magnetic nanoparticles (Miltenyi) for 15 min at room temperature. The cell suspension was then passed through a Pre-separation filter and MS column (Miltenyi), which retained cells bound to Thy1-magnetic beads within the column magnetic field. After rinsing with HABG media, the remaining cells were flushed with 1 ml HABG media. In preparation for FACS, 2 μl/ml of the DNA intercalating dye 7-AAD (BD Pharmingen) was added to the solution to mark cells with compromised cell membranes.

### FACS

The RGC-enriched cell suspension obtained from the immuno-affinity step was passed through a FACS Aria (BD Biosciences) electrostatic sorter. Dead and dying cells were excluded based on high deep red 7-AAD fluorescence. We also excluded cellular debris, which registered as being essentially non-fluorescent, with relatively lower forward light scatter (FSC; an indication of particle size) and side light scatter (SSC; granularity) compared with RGCs.

**Figure 2.**
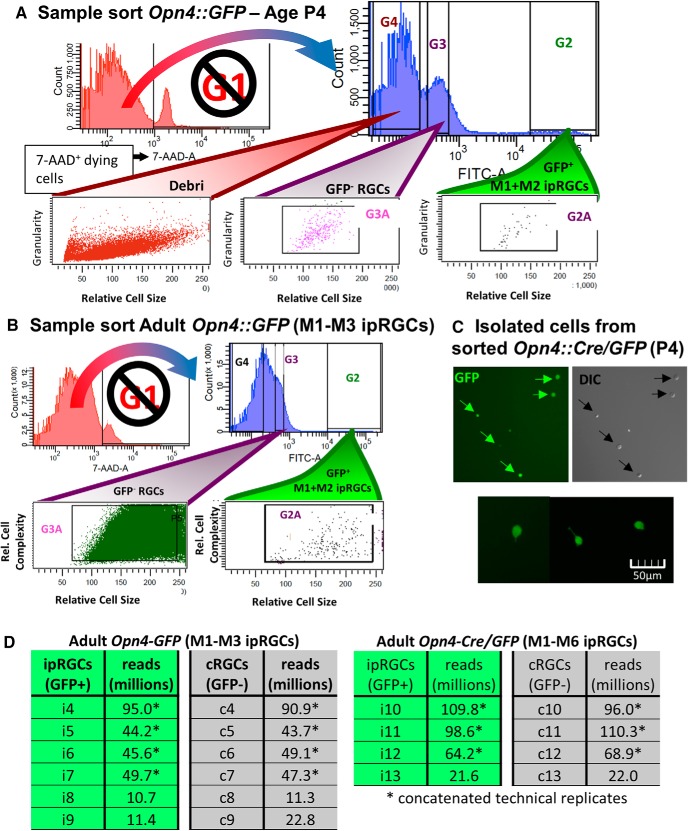
FACS gating strategy for isolating ipRGCs (GFP+) in parallel with GFP-negative cells that are enriched for RGCs. ***A***–***C***, Healthy cells were selected against death marker 7-AAD (not G1). The ipRGCs (GFP^+^) and generic RGCs (GFP^−^) cells were selected based on intensity and similar relative cell size ultimately using gates G2A and G3A, respectively. ***A***, Example sort from retina of P4 *Opn4-GFP* mouse. ***B***, Example sort from retina of young adult *Opn4-GFP* mouse, with noticeably higher debris and cell death. ***C***, Microscopy testing of accurate sorting of GFP+ cells isolated from P4 *Opn4-Cre/GFP* mouse. ***D***, Total sequenced reads from adult *Opn4-GFP* and *Opn4-Cre/GFP* reporters.

The remaining cells were sorted using FITC gating into GFP-positive cells (presumptive ipRGCs in either reporter mouse we used; Fig. 2) and GFP-negative cells [nearly all expected to be conventional RGCs (cRGCs)]. The goal was to compare the transcriptional profiles of these two matched samples to identify genes differentially overexpressed or underexpressed in ipRGCs. The cRGC and ipRGC samples were treated with the same reagents, cytometer settings, centrifugation forces, and temperatures throughout the procedure. cRGCs and ipRGCs had similar SSC and FSC values.

### RNA extraction and cDNA preparation

RNA-processing was done in an enclosed RNase-free environment to limit degradation of RNA throughout the extraction process. FACS-acquired cells were sorted directly into Qiagen RLT buffer with 10 μl/ml of β-mercaptoethanol for immediate lysis. While sorting, the lysis solution was kept at 4°C and periodically mixed. After sorting, we extracted the RNA from the lysed cells using the RNeasy Micro Kit and RNeasy Minelute columns (Qiagen). The enriched RNA was treated on-column with RNase-free DNase I (RNeasy Micro Kit) to remove any residual genomic DNA from the sample. For RNA elution, 12 µl of RNase-free water was added directly to the center of the spin column membrane and centrifuged at >8000 rcf for 1 min. Five microliters of eluted RNA solution was retained for Nugen Ovation cDNA amplification (Caretti et al., 2008; Clément-Ziza et al., 2009; Morse et al., 2010; Tariq et al., 2011). Additionally, integrity of eluted RNA was assessed using PicoChip RIN analysis and RNA amount using the Qubit RNA assay. Initially, we proceeded immediately with cDNA processing after RNA extraction. However, freezing at −80°C did not alter RNA integrity because the frozen RNA samples still received RIN score of 9.0 or greater. Therefore, most of the cDNA libraries were prepared after storage of extracted RNA at −80°C.

### RNA-seq library preparation and sequencing

Before preparing sequencing libraries, we first sheared cDNA to the appropriate size (200–300 bp median) using the Covaris system. Each sample was subsequently processed using Illumina TruSeq kit using a unique bar code adapter to allow for multiplexing multiple samples. Excess adapter sequences were removed using Ampure bead isolation, which removes all DNA fragments <200bp. The library fragment size distribution was tested using High Sensitivity Bioanalyzer and qPCR analysis using primers that match the library adapters (Quail et al., 2008; Fig. 2*D*). Finally, 50 bp single-end sequencing was completed using HiSeq 2000 (∼200 million reads divided among sequencing samples). Initially, we processed eight samples per lane with multiplexed sequencing. We later ran many of the sequencing libraries again with less multiplexing, providing increased sequencing depth. The corresponding technical replicates were merged together for differential expression analysis. The final read counts of each sample are shown in Fig. 2*D*.

**Figure 3.**
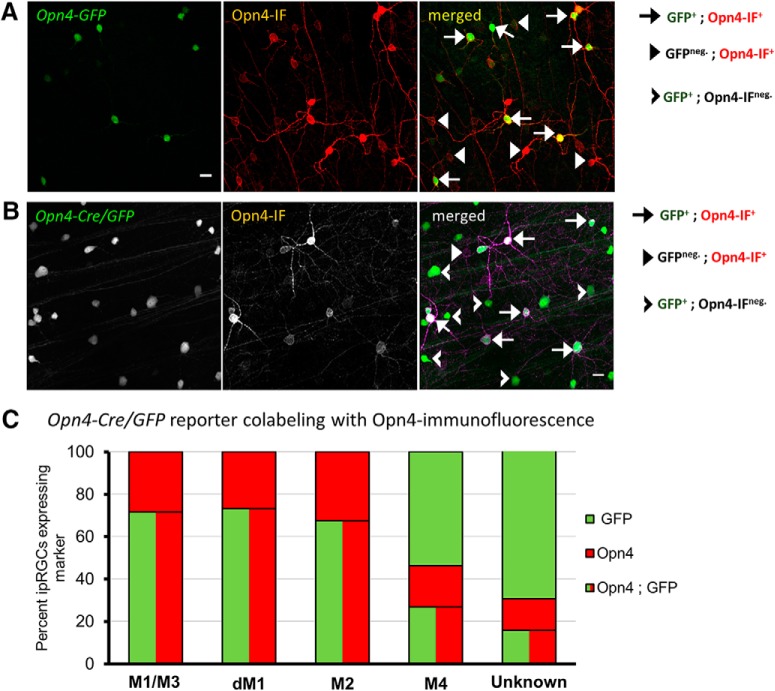
Characterization of Opn4-based fluorescent reporters for gene expression studies. ***A***, Immunofluorescence of anti-Opn4 immunofluorescence (IF) of whole-mount retina from transgenic *Opn4-GFP* mice with fluorescent protein expression in ipRGCs. Red, Opn4-immunolabeling; green, fluorescently labeled cells; yellow, merged colocalized labeling pattern. Scale bar, 20 μm. ***B***, Coexpression of *Opn4-Cre/GFP* labeling with immunofluorescence of anti-Opn4 staining of whole-mount retina. Red, Opn4-immunolabeling; green, fluorescently labeled cells; yellow, merged colocalized labeling pattern. Scale bar, 20 μm. ***C***, Quantification of labeling efficiency of Opn4-immunolabeled M1–M3 ipRGCs by *Opn4-Cre/GFP*. Additional comparison of GFP-labeling in low Opn4-expressing ipRGC subtypes M4 (large soma) and M5/6 (small soma).

### Differential gene and transcript expression analysis

For the tens of millions of reads generated by sequencing we first removed adapter sequences and low-quality reads using fastx_clipper and fastq_quality_filter, respectively. Second, using tophat2 we aligned reads to the mm9 mouse reference genome. Third, the aligned reads were converted to SAM format for htseq-count, which counted the number of reads that aligned to an annotated gene. Finally, we used the EdgeR package to perform statistical analysis on the generated count table to identify quantitative differences in expression levels between the two experimental samples (Anders and Huber, 2010; Trapnell et al., 2012; Anders et al., 2013). EdgeR compares and retains the relationship between all pairs of experimental samples when calculating differential expression likelihood (Anders et al., 2013). Our analysis filtered out genes with very low counts, <1 count per million (cpm), in more than half of the samples used in the differential expression analysis.

### Identification of differentially expressed genes

To identify the set of differentially expressed genes (DEGs) in the ipRGC populations, we used the following strict criteria. First, we identified genes with low false discovery rate (FDR; <0.05) and high fold-change (>2-fold) suggesting differential expression between ipRGCs and generic RGCs. Second, we considered whether the differentially gene expression was corroborated across both reporters (*Opn4::GFP* labeling M1–M3 cells and the *Opn4::Cre/GFP* system that labels M1–M6 ipRGCs) and both ages [P5 (± 1day) or young adult (P30 ± 3 days)]. Third, we identified whether the DEGs have nearly absent gene expression in cRGC samples to distinguish potential for selective gene expression in ipRGCs. This was distinguished both at the level of count-values and manual inspection of aligned raw reads using the Integrated Genome Viewer (IGV; Thorvaldsdóttir and Robinson, 2013). Using IGV, we verified that the reads align with reference gene model for full-length coverage across multiple ipRGC replicates and that there were absent or partial reads aligned across the generic RGC replicates.

Determining differentially repressed genes in adult ipRGCs was confounded by the high amount of contaminants in generic RGC populations. We could not decipher whether a gene with relatively low expression in ipRGCs was the result of non-RGC populations contaminating the generic RGC control population. In contrast, the P5 generic RGC samples from the *Opn4-GFP* reporter were determined to have greatly reduced levels of contamination and similar levels of RGC marker expression. This made it possible to identify genes more weakly expressed in ipRGCs than in generic RGCs in early postnatal development.

### Accession of RNA-seq data

Deposited in NCBI GEO with accession number GSE118780. To review the GEO accession, see https://www.ncbi.nlm.nih.gov/geo/query/acc.cgi?acc=GSE118780.

### Retina tissue preparations and solutions

Mice were killed by inhalation of CO_2_. Before removing the eye, the dorsal margin of the cornea was marked with a cautery and this was used to guide the placement of a large relieving cut in the dorsal retina as a subsequent guide to retinal orientation. Eyes were removed immediately after death and placed in Hibernate-A solution preheated to 37°C. To keep track of retinal orientation, the right and left eye were identified and processed separately.

### Immunohistochemistry

The following primary antibodies were used for our immunofluorescence colabeling studies: rabbit anti-melanopsin (Advanced Targeting Systems; 1:10,000), guinea pig anti-RBPMS (PhosphoSolutions, 1832-RBPMS), rabbit anti-GFP (Invitrogen); Goat anti-Brn3b antibody (Santa Cruz Biotechnology, sc-6026); mouse anti-Rasgrp1 (Santa Cruz Biotechnology, sc-8430); guinea pig anti-Tbx20 (1:8500; Song et al., 2006). Secondary antibodies consisted of AlexaFluor 350, 488, 594, or 647 donkey anti-goat, AlexaFluor 594 donkey anti-rabbit, and AlexaFluor 594 goat anti-guinea pig (Invitrogen or Jackson ImmunoResearch).

For immunofluorescence studies, the dissected retina was flattened onto Millipore nitrocellulose paper after making four small relieving cuts and fixed for 30 min at room temperature (freshly prepared 4% paraformaldehyde in 0.1 m PBS, pH 7.4). The tissue was then washed for 15 min in PBS three times, and then incubated in a blocking solution of 0.5% Triton-X and 5% goat serum in PBS for 2 h at room temperature. The tissue was incubated in the primary antibodies diluted in this same blocking solution for 2 d at 4°C on a shaker. The following day, the samples were washed six times for 20 min in 0.1% Tween 20 in PBS. The tissue was then incubated for 2 h in the appropriate secondary antibodies diluted 1:1000 in the blocking solution at room temperature. The tissue was then washed six times for 10 min in 0.1% Tween 20 in PBS. The retinas were mounted in Aquamount, coverslipped, and sealed with fingernail polish.

For Rasgrp1 immunofluorescence studies that did not include Tbx20 immunofluorescence, an additional antigen retrieval step was included by placing fixed tissue in Tris-EDTA, pH 8.0, for 30 min at 80°C. The samples were then allowed to return to room temperatures (∼15–30 min) before they were removed from the Tris-EDTA solution and washed three times for 15 min in PBS.

Anti-GFP immunofluorescence using AlexaFluor 488-labeled secondary antibody was included for all studies exploiting GFP labeling.

### Image acquisition

Immunofluorescent images were captured on a Zeiss Confocal (LSM 510) and Nikon Eclipse microscope (Micro Video Instruments, E614) with a built in Spot Camera (Diagnostic Instruments, HRD 100-NIK). Confocal images were taken with a 20× objective (Plan Apochromat, WD 0.55 mm) at a resolution of 2048 pixels. To enhance clarity, image files were pseudocolored and the brightness and contrast was adjusted using ImageJ 1.47 (National Institutes of Health). Final images were assembled in ImageJ and PowerPoint (Microsoft).

### Retrograde axon-transport labeling of ipRGCs from the suprachiasmatic nucleus

To label ipRGCs sending axon terminals to the SCN, we used a retrograde tracing method as previously described (Estevez et al., 2012; Stabio et al., 2018). Wild-type mice (4–8 weeks old) were anesthetized by inhalation of 3% isoflurane and placed in stereotaxic apparatus. A craniotomy was performed above the injection site (SCN: −0.5 AP, −5.6 DV, 1.25 ml) and a glass micropipette attached to a Picospritzer II (Parker Hannifin) was used to deliver 200 nl of retrograde tracer rhodamine latex microspheres (RetroBeads, Lumafluor). Three to 5 d later, the brain was removed and immediately fixed overnight at 4°C in 4% paraformaldehyde freshly prepared in 0.1 m PBS. The following day, the brain was rinsed in 0.1 m PBS and sectioned at 50 μm in the coronal plane. The slices were incubated in fluorescent DAPI stain to facilitate histologic identification of the SCN. Fluorescence imaging allowed us to visualize the injection site (rhodamine channel) in relation to the SCN, discernable from UV DAPI fluorescence. For this, we used a SPOT RT Slider digital microscope camera mounted to a Nikon (Diagnostic Instruments) as described previously (Berson et al., 2010; Estevez et al., 2012). Images were assembled in Adobe Photoshop CS3. For all data presented, injections spared the underlying optic chiasm and tracts, thus avoiding nonspecific retrograde labeling of RGCs.

## Results

For our transcriptomics studies, we enzymatically dissociated retinas from melanopsin-reporter mice, selected for RGCs by anti-Thy1 immunoaffinity, and sorted these into presumptive ipRGC and cRGC pools by FACS based on the fluorescent labeling of ipRGCs (Fig. 1*B*). We then compared gene expression in these ipRGC-enriched and cRGC-enriched cell samples to identify genes differentially expressed in ipRGCs compared with other ganglion cells.

We used two strains of melanopsin-reporter mice for these experiments. One of these was a BAC transgenic mouse generated by the GENSAT project (here termed *Opn4-GFP*). Because the GENSAT *Opn4-GFP* reporter line has not been characterized previously, we using anti-melanopsin immunofluorescence to relate the pattern of GFP labeling to that obtained. The M1 and M2 ipRGCs subtypes (and the bistratified M3 variant) express enough melanopsin throughout their somas and dendrites to permit M1 and M2 cells to be differentiated based on their dendritic stratification in the IPL (OFF sublayer for M1 cells; ON sublayer for M2 cells; Berson et al., 2010; Schmidt and Kofuji, 2009). M3 ipRGCs, which are relatively rare, stratify in both the ON and OFF sublamina. Documenting this bistratified pattern in such material is labor intensive, so for efficiency, we simply grouped any cell with melanopsin-immunopositive dendrites in the OFF IPL together and refer to this as the “M1/M3” type. To limit bias, identification and classification of ipRGCs based on Opn4-immunofluorescence was completed first and remained hidden from the analyst until labeling of other features were completed.

All *Opn4-GFP*^+^ cells were Opn4-immunopositive (*n* = 60 GFP^+^ cells across 7 regions of 1 retina; Fig. 3*A*). Approximately one-half of GFP^+^ cells were M1/M3 ipRGCs with somas in the ganglion-cell layer (52.6 ± 6.8% of 66 GFP+ cells across 7 retinal regions), 9.7 ± 5.0% were displaced M1 cells, and the remaining 37.7 ± 3.5 were M2 cells. Unexpectedly, many of the Opn4-immunopositive M1-M3 ipRGCs were not labeled by the reporter. Most, but not all, M1/M3 ipRGCs-immunoreactive for anti-Opn4 also coexpressed GFP (63.3 ± 4.8% of 55 M1 cells), whereas only 28.7 ± 3.9% of M2 ipRGCs (88 cells) were GFP^+^. Therefore, the coexpression of EGFP expression by the *Opn4-GFP* reporter is strongly correlated with M1–M3 ipRGCs, but only accounts for approximately half of the population. In its selective labeling of M1–M3 ipRGCs, this *Opn4-EGFP* reporter resembles another BAC transgenic melanopsin reporter mouse of similar design (Schmidt et al., 2008). The selectivity presumably results from the fact that these ipRGC subtypes express the most melanopsin, and GFP expression is proportionate.

The other reporter mouse used in this study, *Opn4^Cre/+^; Z/EG* mice (*Opn4-Cre/GFP*), has been previously demonstrated to label with EGFP all six known morphologic types of ipRGCs, named M1–M6, while labeling few if any cRGCs (Ecker et al., 2010; Estevez et al., 2012; Stabio et al., 2018; Quattrochi et al., 2019). M4–M6 cells have lower levels of melanopsin-expression than M1–M3 cells and their dendrites are not revealed by Opn4 immunostaining (Ecker et al., 2010; Stabio et al., 2018; Quattrochi et al., 2019). Nonetheless, M4 cells are recognizable among GFP+ cells in this mouse line from their large soma size, and weak melanopsin or absent anti-melanopsin immunolabeling (Estevez et al., 2012).

Opn4-immunofluorescence revealed that more than one-fourth of ipRGCs of the M1, M2 and M3 cells lacked discernable GFP-labeling in this reporter mouse (M1/M3 cells: 28%, *n* = 81; displaced M1 cells: 27%, *n* = 15; M2 cells: 33%, *n* = 132; 4 sampled regions from one *Opn4-Cre/GFP* retina; Fig. 3*B*,*C*). Among presumed M4 cells (large somas; faint Opn4-immunoreactivity) only approximately one-quarter were GFP-positive (27% Opn4^+^;GFP^neg.^, *n* = 67 M4 cells). Other presumed M4 cells (large GFP+ somas) were Opn4-immunonegative (54%, *n* = 67 M4 cells; Fig. 3*C*). GFP^+^ cells with small somas and no evident Opn4-immunoreactivity were designated as “unknown” ipRGC types (72%, *n* = 202 unknown cells; Fig. 3*C*). These were presumably mainly M5 and M6 cells, but this could not be confirmed from dendritic immunostaining. Additionally, we observed small cell bodies of unknown type with weakly Opn4-immunlabeling that did not extend to the dendrites (28%; Fig. 3*C*).

### Cell composition and purity of isolated ipRGCs and cRGCs

**Figure 4.**
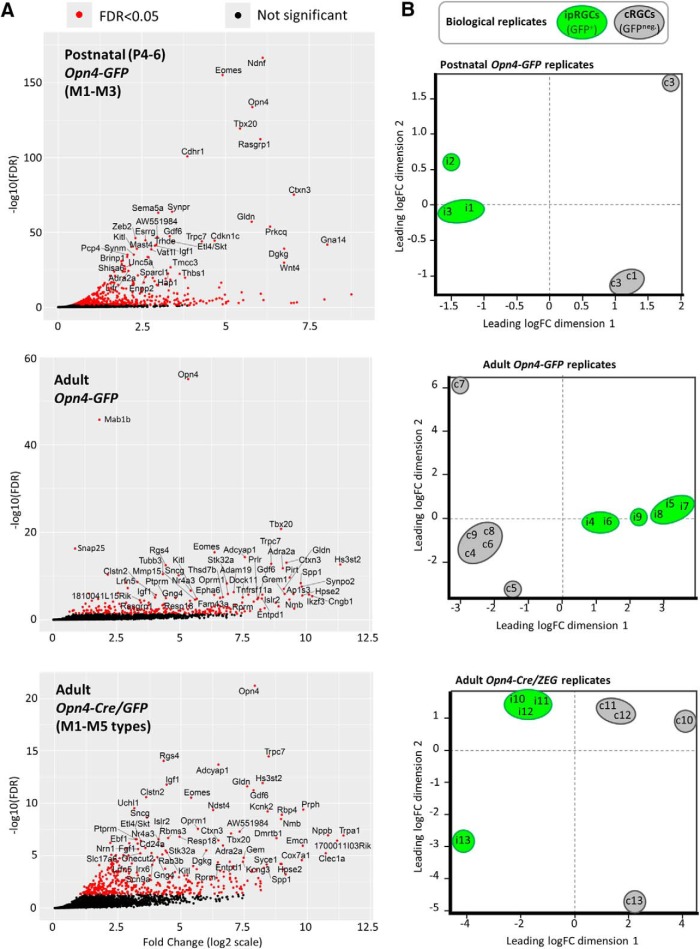
Scatterplot analysis of relative gene expression and dispersion of biological replicates from positively-selected ipRGCs and enriched cRGCs. ***A***, Volcano plot analysis of gene transcripts with positive fold-change (*x*-axis; ipRGC-enriched relative to cRGCs) plotted against the negative logarithm of the FDR (*y*-axis). Significant differentially expressed transcripts (FDR < 0.05) are represented as red dots, whereas transcript with FDR > 0.05 have black dots; blue transcripts, Parv-Cre/TdT enriched; red, SNS-Cre/TdT enriched, twofold, *p* < 0.05). The most significant genes from each sample set are attributed with gene name labels, with limits implemented for text readability (FDR < 1E−20 and logFC > 2 for postnatal age Opn4-GFP reporter; FDR < 1E−4 and logFC > 3 for adult ipRGC reporters). ***B***, EdgeR MDS plot illustrates the overall similarity between expression profiles of different samples. Each sample is denoted by a letter (“i” for ipRGCs; “c” for cRGCs) and a number, corresponding to particular replicate, comprising one pool of purified RGCs then divided into the two pools. Numbering scheme represents paired ipRGCs (GFP^+^) and cRGCs (GFP^−^) replicates (i.e., ipRGC sample “i1” was processed in parallel with cRGC sample “c1”, sample “i2” with “c2”, etc.). Distances are approximately the log2 fold-changes between samples. Green and gray ovals represent ipRGC (GFP^+^) and cRGC (GFP^−^) samples, respectively. Adapted from EdgeR simple graphical output of individual samples in 2D space.

The transcriptional data obtained offered broad internal evidence for the efficacy of purification of cell samples. As expected, the *Opn4* (melanopsin) gene was among the genes much more highly expressed in ipRGCs than in cRGCs. For example, *Opn4* was enriched 40-fold in adult ipRGCs purified from *Opn4-GFP* mice, and this was highly significant, at *q* < 1 × 10^−55^ FDR. Though *Opn4* expression was detected in cRGCs at modest levels (Fig. 4*A*), this was expected, because some ipRGCs lack GFP expression in both melanopsin reporter lines (Opn4-GFP and Opn4-cre/GFP), and these would have been pooled with cRGCs during the FACS procedure.

Transcripts of other genes known to be expressed in ipRGCs were also enriched in the ipRGC pool relative to the cRGC pool (FDR < 0.05, significantly expressed in ipRGC samples, and absent or weakly expressed in cRGC samples). Among these genes were *Adcyap1* (pituitary adenylate-cyclase activating polypeptide; PACAP), *Tbr2* (*Eomesodermin*), *Trpc7* and, to a lesser extent, *Trpc6* (Hannibal et al., 2004; Xue et al., 2011; Sand et al., 2012; Mao et al., 2014; Sweeney et al., 2014; Fig. 4*A*). Together, these results demonstrate that mRNA isolated from purified ipRGC samples were enriched as expected for transcripts for genes that are known to be differentially expressed in ipRGCs.

The relationship among the transcriptional profiles of ipRGC and cRGC samples across replicates are illustrated in the multidimensional scaling (MDS) plots of Figure 4*B*. These show the relationship between all pairs of samples (one of ipRGCs, the other of cRGCs) based on a count-specific pairwise distance measure (Anders et al., 2013; Fig. 4*B*). These sample pairs were clearly separated along the first dimension, indicating pronounced differences in overall gene-expression patterns between ipRGC and cRGC samples. Samples of ipRGCs and cRGCs derived from the same retina and processed in parallel tended to be closely spaced along the second dimension, indicating greater similarity within than across replicates. This may reflect slight differences in overall genetic makeup of mice contributing to each pool, because both strains used were on a mixed genetic background, or to slight technical differences in the acquisition and processing of RNA from one run to the next.

**Figure 5.**
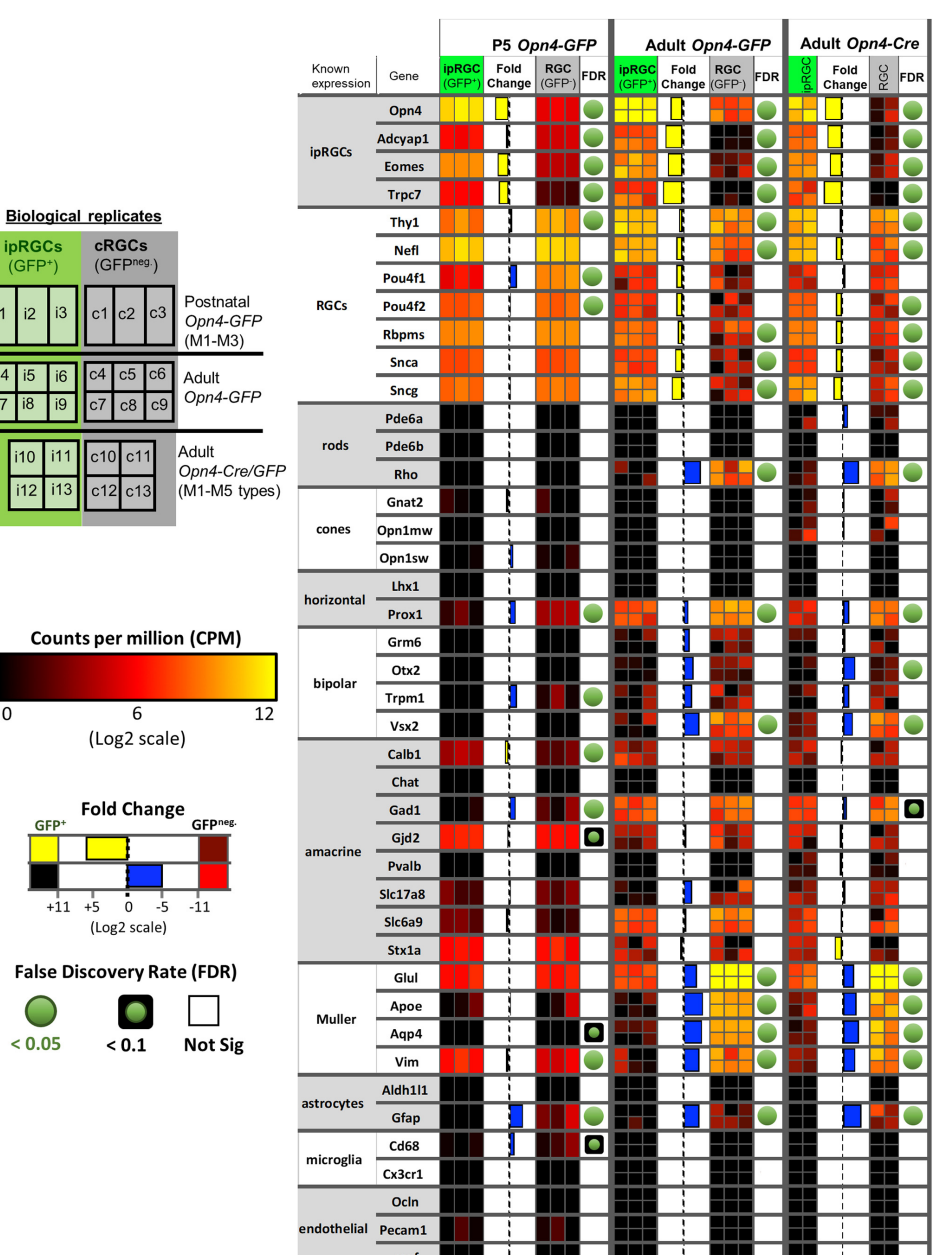
Purity and cell composition assessment of ipRGC and generic RGC samples. Heat map of known cell type marker gene expression in the retina to assess purity and cell composition of ipRGC and generic RGC samples. Shown are biological replicates tested for *Opn4-GFP* (P5 and adult) and *Opn4-Cre/GFP* reporters. Relative expression levels, fold-change, and FDR are color-coded as indicated in the figure. White boxes indicate high gene expression, whereas blue represents little or no detected expression. FDR is not available (NA) in cases that our analysis filtered out genes with very low counts, <1 cpm, in more than one-half of the samples used in the differential expression analysis.

In the purified ipRGC samples, we found little or no evidence of contamination by transcripts from other retinal cell types. For example, transcript levels were very low for rod and cone opsins, for the amacrine-specific marker ChAT, for several bipolar markers (Otx2; Vsx2; Grm6; Trpm1), and for markers of astrocytes, microglial and vascular cells (Fig. 5). Several transcripts suitable for assessing potential contamination from Müller glia (*Glul*, *Vim)* were present at surprisingly high levels in the purified ipRGC samples, suggesting that these glial cells may contaminate the transcriptional picture to some degree.

In general, the cRGC samples were relatively less pure than the ipRGCs samples by this measure. A particularly informative transcript for assessing such contamination is that for the rhodopsin gene (*Rho*), because rods are by far the most common neuronal type in the mouse retina and express *Rho at* very high levels. Rhodopsin transcripts were significantly (150-fold) more abundant in the cRGC samples than in ipRGC samples (Fig. 5), whether isolated from *Opn4-GFP* or *Opn4-Cre/GFP* adult reporter mice (FDR < 6 × 10^−9^). Evidently, the second isolation step in which GFP+ positive cells were isolated by FACS from the purified RGC pool was a key factor in the greater purity of the ipRGC sample. Similarly, transcripts associated with bipolar cells and Müller glial cells were generally more abundant in cRGC than ipRGC samples. For example, the cRGCs had relatively high expression of the known Müller glia markers *Glul*, *Apoe*, *Aqp4*, and *Vim*, generally higher than in the ipRGC pool (Fig. 5). Contamination of adult cRGC samples by other cell types may explain why most RGC markers, such as *Rbpms* and *Sncg* (Soto et al., 2008; Rodriguez et al., 2014), were less abundant in the cRGC cell pool than in the ipRGC pool. However, the data suggest that contamination in the cRGC pool was not uniform across retinal cell types. Amacrine-specific transcripts were no more abundant overall in cRGCs than in ipRGCs, and microglial and vascular markers were essentially absent, as in ipRGCs.

In immature mice (P5; *Opn4-GFP*), contamination of cRGC samples by non-RGC transcripts appeared more modest than in adults. The major sources of contamination (rods and Müller glia) are still being born and undergoing early-stage differentiation at this age, and this would presumably depress the abundance of their cell-type-specific transcripts (Young, 1985; Morrow et al., 1998; Matsushima et al., 2011).

To summarize, this analysis suggests that the ipRGC samples were relatively free of contamination by most other retinal cell types. In contrast, contamination of the cRGC samples appears to derive mainly from Müller cells and strongly expressed genes in rods. Though this must be factored into the analysis, our primary focus was on genes more strongly expressed in ipRGCs than in cRGCs, and this difference seems unlikely to be affected by the modest contamination of the cRGC pool.

### Genes differentially expressed in ipRGCs

**Figure 6.**
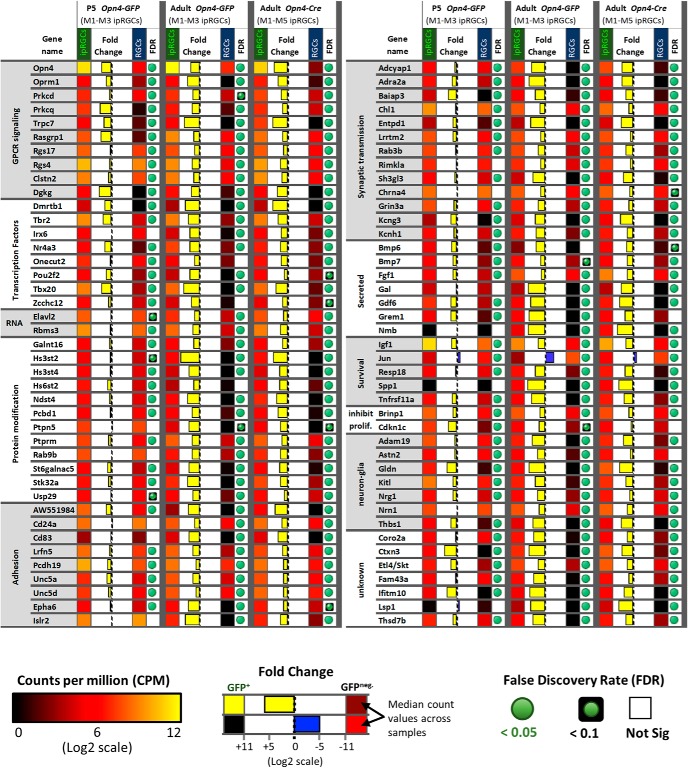
The expression pattern of candidate ipRGC-specific genes. Heat map of 83 genes differentially expressed in ipRGCs that have functional links to GPCR signaling, regulation, and maintenance of molecular programs, neuron communication and organization, neuron survival, and neuron-glia interactions. Relative expression levels, fold-change, and FDR are color-coded as indicated in the figure.

Comparing the abundance of transcripts in the ipRGC and cRGC pools, we identified >75 genes that were differentially elevated expression in ipRGCs (as marked by one or both melanopsin-reporter lines) relative to cRGCs. Briefly, identification of DEGs in ipRGCs relied on the following stringent criteria: (1) low FDR with high fold-change, (2) corroboration of differential expression across both ipRGC reporters, and (3) absence of gene expression in cRGC samples (see Materials and Methods). Some instances of genes with borderline FDR in a subset of samples, such as *Zcchc12* and *Hs6st2*, were included after closer manual inspection of aligned reads determined relative high expression in ipRGCs combined with absent or relatively low aligned reads in cRGC samples. The identified DEGs are diverse, and most have not been previously identified as ipRGC-enriched (Fig. 6; see Materials and Methods). Here, we survey some of these genes, grouped by their functional features (Fig. 6).

#### Expression differences between the *Opn4-GFP* and *Opn4-Cre/GFP* reporter systems

**Figure 7.**
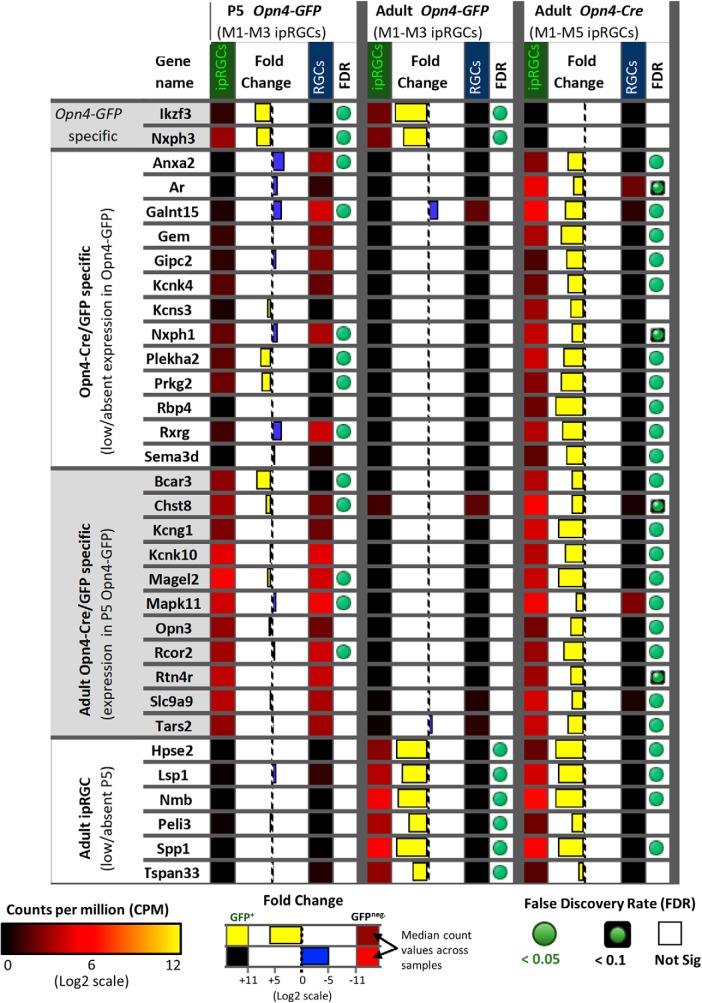
Expression pattern differences between Opn4-based reporters. Heat map of genes differentially expressed in adult ipRGCs labeled by the *Opn4-Cre/GFP* reporter (M1–M6 ipRGCs) compared with *Opn4-GFP* (M1–M3 ipRGCs). Relative expression levels, fold-change, and FDR are color-coded as indicated in the figure.

To study gene expression differences across the different ipRGC subtypes, we compared the expression patterns of *Opn4-Cre/GFP* (labels M1–M6 subtypes) and *Opn4-GFP* (labels only the M1–M3 subtypes; Fig. 7). In general, genes differentially expressed in ipRGCs identified in the two reporter systems were both supportive and cross-correlated. However, we identified 24 genes that were differentially expressed in the adult *Opn4-Cre/GFP* reporter but had low or no apparent expression in the *Opn4-GFP* reporter, suggesting selective expression in one or more of the M4–M6 ipRGC subtypes. The *Opn4-Cre/GFP*-specific genes included *Anxa2*, *Gem*, *Sema3d*, *Rbp4*, and *Rxrg*. Recently, an Rpb4 reporter (Rbp4-Cre) was demonstrated to mark amacrine cells coupled to ipRGCs, although there was an apparent lack of labeling in ipRGCs (Sabbah et al., 2017). The Kcnk4/TRAAK, another gene that was differentially expressed in the *Opn4-Cre/GFP* reporter, encodes a two-pore potassium channel subunit (Fink et al., 1998). Additionally, our data suggest that the Kcns3 electrically silent voltage-gated potassium channel subunit has its expression restricted to the ipRGCs labeled by *Opn4-Cre/GFP*, but this difference did not reach statistical significance (FDR 0.13). However, close inspection of reads aligning to Kcns3 using the IGV confirmed weak expression in ipRGCs and absent expression in cRGCs for *Opn4-Cre/GFP* samples (data not shown). Last, the neurexophilins Nxph1 and Nxph3 were differentially expressed in the *Opn4-GFP* and *Opn4-Cre/GFP* reporters, respectively (Fig. 7). These proteins are known to bind α-neurexins in mice and have restricted expression patterns (Missler et al., 1998; Beglopoulos et al., 2005; Craig and Kang, 2007).

#### Transcription factors

Transcription factors, by regulating other genes, help to generate and maintain ipRGC identity. We noted above that the T-box transcription factor *Tbr2* was much more strongly expressed in adult ipRGCs than in cRGCs, as expected (Sweeney et al., 2014). *Tbr2* is best known for its key role in early retinal development. Its expression in adult retina is far more restricted, but it remains expressed in the majority of ipRGCs. A second T-box transcription factor, *Tbx20*, was similarly enriched (Fig. 6). *Tbx20* has not been previously linked to adult retinal function, but we will show that it too is quite selectively expressed in ipRGCs. Additionally, four other transcription factors, *Irx6*, *Dmrtb1*, *Nr4a3*, and *Pou6f2,* were differentially expressed in adult ipRGCs. Most of these genes serve as broad lineage determinants in early retinal development (Zhou et al., 1996; Star et al., 2012). Other highly expressed genes included the neuron-derived orphan receptor 1 *Nor1* (*Nr4a3*), which codes for a nuclear receptor, and *Elavl2* gene, which codes for a RNA-binding protein important for mRNA metabolism and neuronal differentiation (Fornaro et al., 2007; Hinman and Lou, 2008). Pathway analysis (DAVID) of DEGs in ipRGCs suggested specialization in heparan sulfate biosynthesis, including *Hs3st4*, *Hs3st2*, *Hs6st2*, *Ndst4*, and *Gpc5* (Fig. 6).

#### Receptors and channels

**Figure 8.**
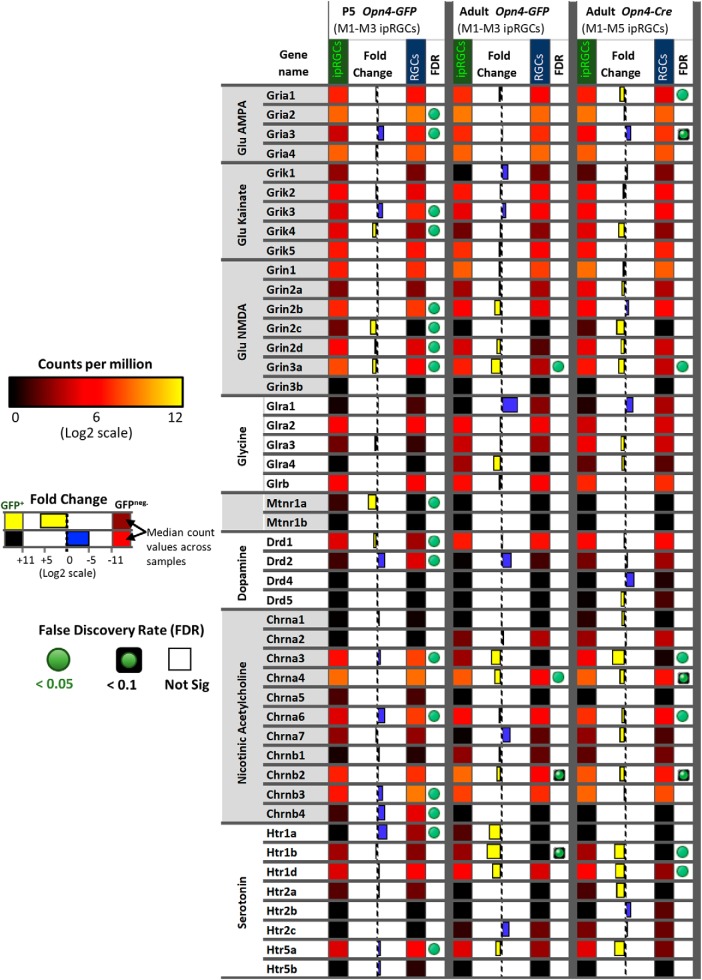
The expression pattern of neurotransmitter receptors. Heat map of genes encoding for nicotinic acetylcholine, dopamine, serotonin, glycine, glutamate, and melatonin receptors. Relative expression levels, fold-change, and FDR are color-coded as indicated in the figure.

Multiple genes encoding diverse surface receptors were differentially expressed in ipRGCs (Fig. 8). For example, expression data suggest that ionotropic nicotinic acetylcholine receptors in ipRGCs may be composed of α3, α4, α6, β2, and β3 subunits (Fig. 8), although the α3 and α4 transcripts were borderline for differential expression in ipRGCs. In agreement with previous studies, we found that ipRGCs expressed the Drd1 dopamine receptor, but had low levels of Drd2 expression (Van Hook et al., 2012). Several serotonin receptor genes (Htr1b, Htr1d, and Htr5a) were modestly enriched in ipRGCs. The ipRGCs were also found to express many glutamate receptors subunits, but only one of these, the NMDA receptor subunit 3A (GRIN3A), was differentially expressed relative to other adult RGCs. The mu opioid receptor gene *Oprm1* is differentially expressed in ipRGCs; it could regulate their light responses interacting with L-type calcium channels, which carry the majority of light-evoked inward calcium current in ipRGCs (Moises et al., 1994; Diaz et al., 1995; Doğrul et al., 2001; Hartwick et al., 2007). Recently, μ-opioid receptors (MORs) immunoreactivity was identified on rodent M1–M3 ipRGCs and MOR activation reduced ipRGC excitability via modulation of voltage-gated potassium and calcium currents (Cleymaet et al., 2019). Our data appear at odds with earlier reports that M1 and M4 ipRGCs express the melatonin receptor genes Mtnr1a and Mtnr1b (Sengupta et al., 2011; Pack et al., 2015; Sheng et al., 2015). Additionally, *Kcnh1*, also known as ether-a-go-go (*Eag1*), was differentially expressed in ipRGCs (Fig. 6). Kcnh1 is a voltage-gated K^+^ channel that has been shown to be crucial for the generation of dark current in the inner segment of rods (Frings et al., 1998), but may normally regulate other neuronal functions in ipRGCs (Martin et al., 2008).

#### Cell adhesion

**Figure 9.**
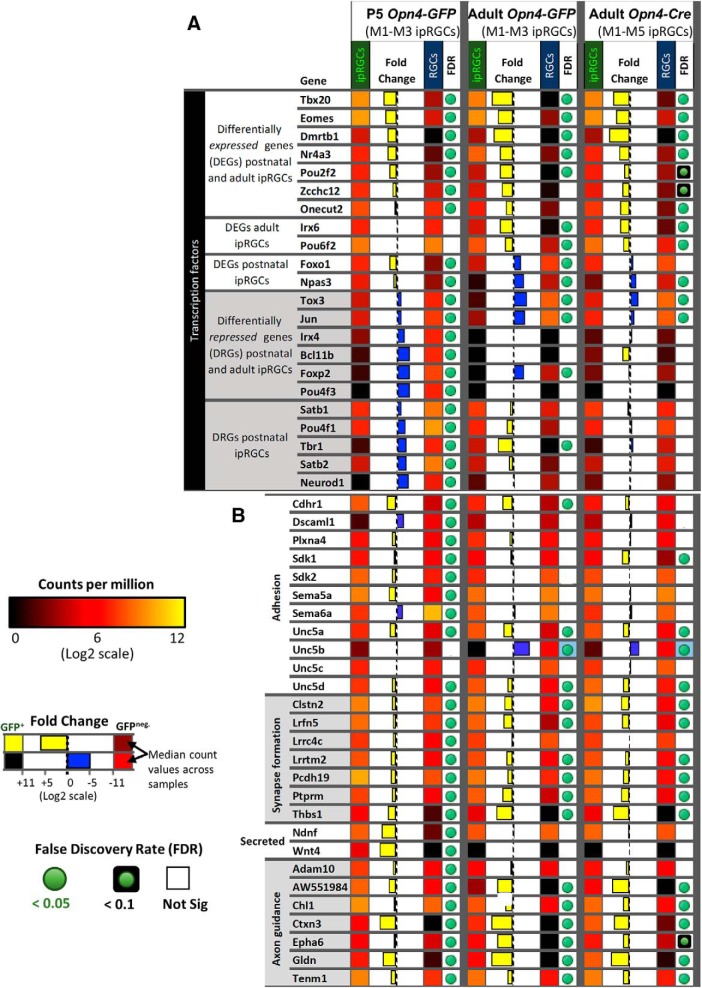
The expression pattern of developmentally regulated genes in ipRGCs. ***A***, Heat map of genes encoding transcription factors that have a particular temporal pattern of differential expression in ipRGCs (e.g., high gene expression in P5 ipRGCs relative to adult expression). Relative expression levels, fold-change, and FDR are color-coded as indicated in the figure. ***B***, Heat map of genes relevant for development of ipRGCs. Relative expression levels, fold-change, and FDR are color-coded as indicated in the figure.

Genes encoding for several cell adhesion molecules were differentially expressed in ipRGCs (Fig. 9*B*). For example, the cell adhesion molecule *DscamL1* was relatively low in ipRGCs during postnatal development, but the closely related genes encoding the Ig superfamily adhesion molecules *Sidekick-1* and *Sidekick-2* were enriched in developing ipRGCs. *Unc5a* and *Unc5d* were significantly differentially expressed both in early postnatal and adult ipRGCs. In contrast, expression of *Unc5b* and *Unc5C* in ipRGCs was low relative to that in cRGCs. As suggested previously, expression of the repulsive ligand *Sema6a* was significantly lower in ipRGCs than cRGCs during postnatal development (Matsuoka et al., 2011). However, its receptor *PlxnA4* was enriched in P5 ipRGCs. Another semaphorin, *Sema5a*, was also significantly enriched in developing ipRGCs. Other differentially expressed cell-adhesion molecules *Salm5* (*Lrfn5*), *Clstn2*, *Thbs1*, *Lrrtm2*, *Pcdh19*, *Ptprm*, and *Lrrc4c (Ngl1)* could play significant roles in the formation of ipRGC synapses (Burden-Gulley and Brady-Kalnay, 1999; Lin et al., 2003, 2018; de Wit et al., 2009; Xu et al., 2010; Lipina et al., 2016; Pederick et al., 2016). The cell surface glycoprotein *Mdga1* was also differentially expressed in developing ipRGCs, and is known to influence the formation and maintenance of inhibitory synapses (Pettem et al., 2013).

#### Tolerance to stress

There is increasing evidence that ipRGCs are resistant to stress and able to survive under circumstances that are fatal for other retinal neurons (Li et al., 2008; de Sevilla Müller et al., 2014; Cui et al., 2015; Duan et al., 2015). The harsh dissociation and FACS processing has the potential of generating stress-induced gene expression changes (Fig. 6). We attempted to identify potential survival molecular programs that are specific to ipRGCs compared with generic RGCs. The genes *Adcyap1* (PACAP), *Igf1*, and *Spp1* (*osteopontin*), all of which have previously described roles in promoting ipRGC survival (Atlasz et al., 2010; Duan et al., 2015) were differentially expressed in ipRGCs. We also identified a number of genes related to glial function differentially expressed in ipRGCs, including *Gldn*, *Cntn2*, *Lama4*, and *Astn2*, and *Thbs1* (Fig. 6).

#### Phototransduction

**Figure 10.**
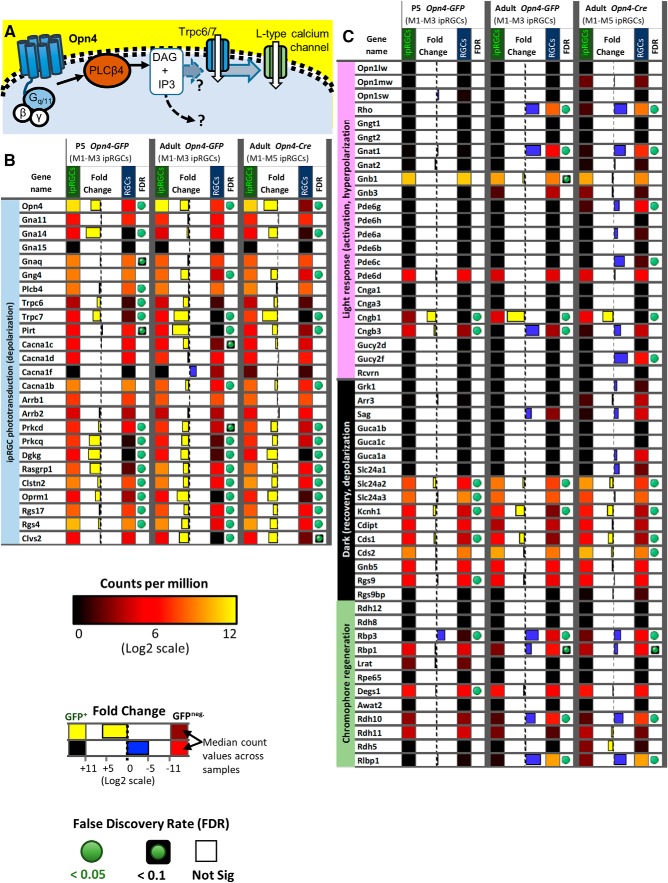
Phototransduction-related gene expression. ***A***, Distinct from rod and cone photoreceptors, the light-activation of Opn4 triggers a membrane-bound signaling cascade including G_q/11_ type G-proteins, the generation of DAG by PLCβ4, the opening of downstream TRPC6 and TRPC7 channels, and ultimately leads to the influx of calcium through L-type voltage-gated calcium channels. ***B***, Heat map of genes that are potentially relevant to the Opn4-mediated phototransduction signaling cascade. Relative expression levels, fold-change, and FDR are color-coded as indicated in the figure. ***C***, Heat map of genes previously described to play a role in the light response, dark adaptation, and chromophore regeneration of rod and cone photoreceptors. Relative expression levels, fold-change, and FDR are color-coded as indicated in the figure.

Photoactivation of melanopsin photopigment typically triggers a phosphoinositide signaling cascade resembling that in rhabdomeric (invertebrate) photoreceptors, involving G proteins in the Gq family, phospholipase C, and canonical TRP channels. In ipRGCs, the phototransduction cascade typically signals through Gq-family proteins and phospholipase C beta 4 (PLCB4) to open canonical TRP channels (Trpc7 and Trpc6; Graham et al., 2008; Xue et al., 2011; Hu et al., 2013; Emanuel and Do, 2015; Emanuel et al., 2017; Fig. 10*A*). Additionally, M2 and M4 ipRGCs use distinct ciliary phototransduction pathway components, including cyclic nucleotide as the second messenger and HCN as the ion channel for phototransduction (Jiang et al., 2018). Further, contrast sensitivity of M4 cells is enhanced by Opn4 phototransduction through the regulation of potassium leak channels (Sonoda et al., 2018).

We determined that the genes in this signaling cascade (*Opn4*, *Trpc7*, *Trpc6*, *Plcb4*, and several Gq genes) were expressed at relatively high levels in all three ipRGC pools (i.e., selective-postnatal; selective-adult; or pan-subtype adult). Moreover, two key genes, *Opn4* and *Trpc7*, were more highly expressed in ipRGCs than in cRGCs in all three ipRGC pools. *Trpc6* was also significantly overexpressed in ipRGCs in younger animals, with a trend in this direction also in adult ipRGCs, but *Trpc7* was expressed at much higher levels than *Trpc6*. Plcb4 appears essential for melanopsin phototransduction in some cells, and it was expressed at much higher levels than Plcb1, 2, or 3. However, Plcb4 was differentially expressed in postnatal age ipRGCs compared with cRGCs, but had similar expression during adulthood.

Recent evidence indicates that multiple the Gα subunits of the Gq family, including Gnaq, Gna11, or Gna14 subunits, redundantly contribute to phototransduction in ipRGCs (Hughes et al., 2015). Our studies suggest a similar expression pattern, including a lack of Gna15 expression (Fig. 10*B*). Further, we determined that Gna14 was differentially expressed in our P5 ipRGC samples, but it did not reach a statistical significant difference in adult Opn4-GFP ipRGCs. Gnaq appears to be among the highest expressing Gq/11 subunits in our study, which conflicts with the negative finding of Siegert et al. (2012). To date, the Gβγ complex involved in the ipRGCs signaling cascade remains unknown. Our studies determined that the beta subunit Gnb1 is by far the most highly expressed in ipRGCs, having a 15-fold higher expression than the other subunits Gnb2, Gnb4, or Gnb5; Gnb3 was not detectably expressed in adult ipRGCs (Fig. 10*C*). Additionally, we found that the gamma subunit Gng4 is differentially expressed in ipRGCs.

Also differentially expressed in ipRGCs were two factors with known roles in diacylglycerol (DAG) signaling, Ras guanyl nucleotide-releasing protein 1 (*Rasgrp1*), and diacylglycerol kinase gamma (*Dgkg*; Fig. 10*B*). *Rasgrp1* is a GEF that activates *Ras* by facilitating its GTP binding (Bivona et al., 2003). Rasgrp1 binds DAG and Ca^2+^, both of which are elevated by melanopsin phototransduction. This provides a possible basis for intrinsic photoresponses of ipRGCs to modulate Ras signaling and thus genes governing cell growth, differentiation and survival. We will return to a more detailed consideration of Rasgrp1 later in this report.

*Dgkg* converts DAG to phosphatidic acid, thus acting as a terminator of DAG signaling (Bivona et al., 2003; Shulga et al., 2011). Because DAG appears to be a key link between early steps in phototransduction and gating of the light-activated channels, Dgkg may regulate the kinetics of the photoresponse in ipRGCs. The protein products of the two overexpressed genes may interact. Diacylglycerol kinases are also known to bind to Rasgrp and modulate its activity (Topham and Prescott, 2001). Diacylglycerol and calcium are also known to activate the protein kinase C (PKC) family members Prkcd and Prkcq (Oancea and Meyer, 1998), which we determined to be differentially expressed in ipRGCs. PKC activity has been suggested to be important for deactivating TRPC activity in the invertebrate photoreceptors and potentially also for the Opn4 phototransduction cascade (Graham et al., 2008). Peirson et al. (2007) previously identified another PKC member, Prkcz, as being important for ipRGC-mediated photoentrainment of circadian rhythms (Peirson et al., 2007). However, Prkcz is only moderately expressed in ipRGCs in our data, at levels and similar to those in cRGCs.

In other photoreceptors, RGS (regulator of G-protein signaling) proteins play a key role in terminating the photoresponse by accelerating the intrinsic GTPase activity of the cognate G-protein (e.g., transducin in rods). Two RGS genes were overexpressed in all three ipRGC pools: *Rgs4* and *Rgs17*. At least one of these (Rgs17) regulates Gq signaling (Mao et al., 2004; Ji et al., 2011; Fig. 10*B*).

The arrestins also contribute to response termination by binding to phosphorylated opsin. ipRGCs exhibited strong expression of both beta arrestin genes (*Arrb1*, *Arrb2*) but low expression of rod (*Sag*) and cone (*Arr3*) arrestin genes. This is consistent with earlier evidence that beta arrestins rather than conventional retinal arrestins bind photoactivated melanopsin in ipRGCs (Cameron and Robinson, 2014; Mure et al., 2018). Still, these beta arrestin transcripts are both at similarly high levels in cRGCs as in ipRGCs, presumably because these arrestins regulate diverse GPCRs (Fig. 10*B*).

Many of the genes involved in rod and cone phototransduction had low expression (scarce or no read alignment) and/or were present at much lower levels in ipRGCs than cRGCs. These include the genes for opsins, transducin alpha, and arrestin in rods (*Rho*, *Gnat1, Sag*) and cones (*Opn1mw, Opn1sw, Gnat2,* and *Arr3*; Fig. 10*C*). Although Cngb1 was differentially expressed in ipRGCs, the total reads aligning to the Cngb1 locus were low and derived mainly from a limited region of the gene, and the obligatory alpha subunits were not detected, so this could be a false-positive (Fig. 10*C*).

### Genes differentially repressed in ipRGCs

The lack of contamination by non-RGC retinal neurons in the P5 samples allowed us to identify genes that were differentially repressed in ipRGCs compared with cRGCs in early postnatal development. Our data suggested that the transcription factor *Jun* (Jun proto-oncogene) and *Irx4* are differentially repressed in P5 ipRGCs samples (Fig. 9). Other genes that were differentially repressed in the P5 ipRGC samples included *Satb1*, *Satb2*, and *Foxp2*, all of which are known to have restricted expression in the abundant F-RGC type that is likely included in the cRGC samples (Rousso et al., 2016). The *Pou4f1* (*Brn3a)* and *Pou4f3* (*Brn3c*) transcription factors were both differentially repressed in P5 ipRGCs, consistent with their known lack of expression in ipRGCs (Jain et al., 2012; Fig. 9). The transcriptional repressors *Bcl11b (CTIP2)*, *Irx4*, and *Tbr1* were all found to be differentially repressed in ipRGC compared with cRGCs samples. Furthermore, the *Cdkn1c* (*p57KIP2),* a gene known to be transcriptionally repressed by Bcl11b (Topark-Ngarm et al., 2006), had relatively increased expression in ipRGCs (Fig. 6).

### Rasgrp1 is selectively expressed in ipRGCs

Because differential mRNA expression does not guarantee a correspondence with protein product (Koussounadis et al., 2015), we sought to test our transcript-level differential expression analysis at the protein level and to determine whether their expression is selective for particular adult ipRGC subtypes. Transcriptional profiling suggested that Rasgrp1 is expressed differentially, possibly even selectively, in ipRGCs. Rasgrp1 has both a calcium and a diacylglycerol binding domain and has well-described role in lymphocytes as a Ras GEF, a nucleotide exchange factor activating Ras through the exchange of bound GDP for GTP activating Erk/MAP kinase. The phototransduction cascade in ipRGCs elevates DAG and intracellular calcium, both of which mediates the Ras GEF activity of Rasgrp1 cascades (Bivona et al., 2003). Therefore, Rasgrp1 is well-positioned to provide a unique form of Ras signaling to a subset of ipRGCs.

**Figure 11.**
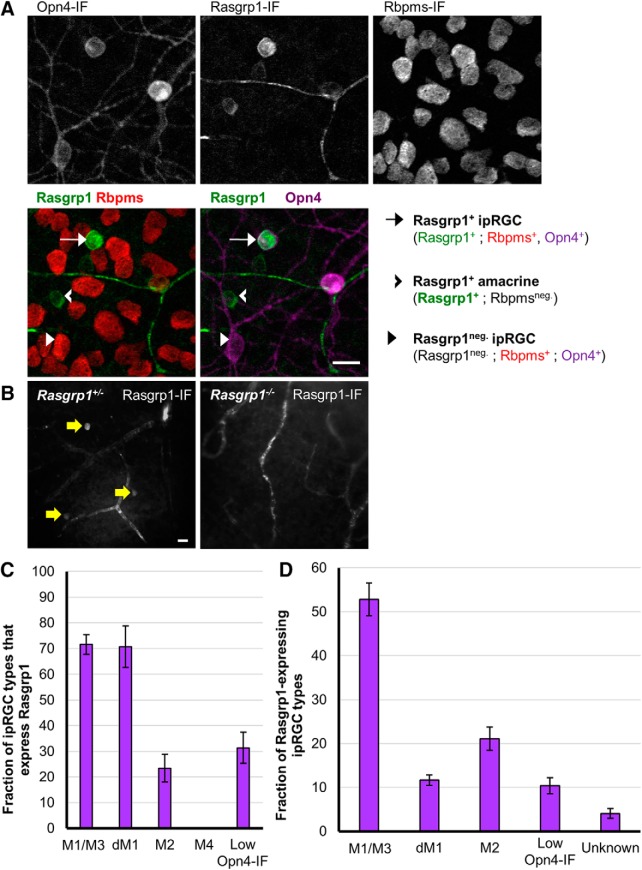
Rasgrp1 is selectively expressed in ipRGCs. ***A***, Whole-mount retina immunostained for Opn4, Rasgrp1, and the pan-RGC marker Rbpms (gray scale). Focal plane is at the GCL. We quantified colocalization of the three markers in confocal images of 49 regions that were topographically dispersed across three whole-mount adult retinas. Colocalization of Rasgrp1 (green), Rbpms (red), and Opn4 (magenta). Rasgrp1 is expressed in a subpopulation of amacrine cells and RGCs (Rbpms-negative and -positive, respectively). Scale bar, 20 µm. ***B***, Rasgrp1 immunolabeling (antibody sc-8430) of cell bodies in GCL of Rasgrp1± heterozygous mice (left, yellow arrows). Absence of cell body immunolabeling in Rasgrp1^-/-^ knock-out mice (right) suggests a lack of cellular off-target antibody staining. ***C***, Quantification of Rasgrp1-expression across Opn4-immunopositive ipRGC subtypes. 70% of M1 and displaced M1 (dM1) cells were Rasgrp1-immunopositive, whereas only 20–30% of either M2, M5, or M6 cells were Rasgrp1-immunopositive. None of the identified M4 cells were Rasgrp1-immunopositive. M1 and M3 types were combined during the process of coexpression analysis (designated M1/M3). Error bars represent SEM. ***D***, Distribution of all Rasgrp1-expressing RGCs (Rasgrp1^+^;Rbpms^+^) that belong to specific RGC types, to the extent that could be determined, including Opn4-immunoreactive ipRGC subtypes. No examples of M4 cells were observed to express Rasgrp1. The vast majority (96%) of Rasgrp1-RGCs are Opn4-immunopositive and therefore ipRGCs. The remaining “unknown” RGC types expressing Rasgrp1 (Rasgrp1^+;^ Rbpms^+;^ Opn4^neg.^) could be a low-expressing ipRGC type or conventional RGCs. Error bars represent SEM.

We used immunofluorescence against Rasgrp1 (Puente et al., 2000) to label the Rasgrp1 protein in whole-mount retinas from adult wild-type mice. Rasgrp1-immunopositive somata were present in the ganglion cell layer (GCL) and in the inner nuclear layer (INL). The latter likely represent amacrine cells or displaced ganglion cells, judging by their close proximity to the inner plexiform layer (IPL; Fig. 11). Immunolabeling marked the cytoplasm as well as the somatic plasma membrane of these cells. Occasionally, particularly strongly Rasgrp1-labeled cells had some dendritic labeling. Rasgrp1 immunostaining was also observed in a subset of photoreceptors in the outer retina (data not shown).

To test whether the Rasgrp1-positive cells in the ganglion-cell layer were RGCs, we conducted double immunofluorescence for both Rasgrp1 (antibody m199) and the RNA-binding protein Rbpms, which selectively labels all and only RGCs (Rodriguez et al., 2014). Approximately one-half of Rasgrp1-immunopositive cells in the GCL were RGCs, as determined by colabeling for Rbpms (56%, *n* = 708 across 3 retinas, 3 mice; Fig. 11*A*). Most of these Rasgrp1-expressing RGCs were ipRGCs, as revealed by their immunoreactivity for melanopsin (95.9 ± 1.1%, *n* = 412; Fig. 11*A*). In contrast, only a fraction of Opn4-immunopositive ipRGCs were Rasgrp1-immunopositive (34%, *n* = 1169). The remainder of the Rasgrp1-immunopositive cells that are immunonegative for Rbpms (non-RGCs), expectedly, lack Opn4-immunoreactivity and can be assumed to be displaced amacrine cells. Thus, Rasgrp1 expression in the GCL is apparently restricted to a subpopulation of ipRGCs and many amacrine cells.

We next tested whether the immunolabeling of RGCs represented endogenous Rasgrp1 protein expression. The antibody used in this study has been previously shown to specifically label Rasgrp1 protein expression in hippocampal neurons (Pierret et al., 2000). As a further test for the specificity of the antibody, we compared immunofluorescence labeling of whole-mount retinas from normal and Rasgrp1-knock-out mice generated by inserting *LacZ* and a *Neo* cassette into the Rasgrp1 gene to disrupt its expression (Dower et al., 2000). Our control experiments showed that the GCL and INL cellular immunolabeling is absent in the Rasgrp1 knock-out (Fig. 11*B*). However, vasculature and photoreceptor cell labeling persisted in Rasgrp1-knock-out mouse retinas, suggesting cross-reactivity of antibody with other proteins.

### Rasgrp1 expression is restricted to diverse ipRGC subtypes

We next determined which of the established morphologic subtypes of ipRGCs express Rasgrp1 into adulthood (Fig. 11*C*). For this purpose, we used key characteristics such as relative Opn4 expression, soma size, and dendritic morphology. In the GCL, the majority of M1/M3 cells (71.6 ± 3.9%, *n* = 300 M1/M3 across 3 retinas, 3 mice), but only a fraction of M2 cells (23.4 ± 5.4%, *n* = 389 M2 cells) expressed Rasgrp1 (Fig. 11*C*). Additionally, many cells with low Opn4-immunofluorescence (designated “low Opn4-IF”) also expressed Rasgrp1 (31.4 ± 6.13%, *n* = 138). Within the INL, displaced M1 cells express Rasgrp1 at a similar percentage as conventionally placed M1 cells (70.7 ± 8.0%, *n* = 3 retinas). We found no examples of Rasgrp1 immunoreactivity in M4 cells (0%, *n* = 172; Fig. 11*C*). Of the Rasgrp1-expressing ipRGCs, half were M1/3 cells (52.8 ± 3.8%), nearly a quarter were M2 cells (21.1 ± 2.7%) and a small percentage (10.4 ± 1.8%) were low Opn4-IF cells (*n* = 396 Rasgrp1^+^/Opn4^+^ cells across 3 retinas, 3 mice; Fig. 11*D*). Therefore, Rasgrp1 is selectively expressed in a diverse set of ipRGC subtypes.

### Tbx20 is expressed in a diverse subset of ipRGCs

**Figure 12.**
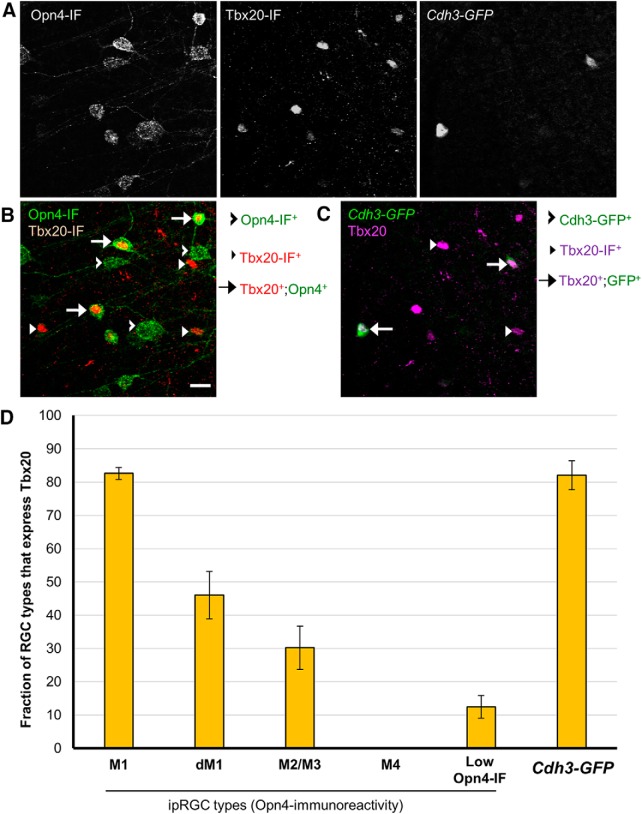
Colocalization study of Tbx20-expression in ipRGC subtypes. ***A***, Triple immunofluorescence of Opn4, Tbx20, and *Cdh3-GFP* (gray scale). ***B***, Tbx20-expression in subset of M1–M3 ipRGCs as well as an additional population of Opn4-immunonegative cells. ***B***, ***C***, Tbx20 is concentrated in the nucleus of most *Cdh3-GFP*-cells. ***D***, Quantification of Tbx20-expression across Opn4-immunopositive ipRGC subtypes. Tbx20 immunofluorescence labels multiple ipRGC subtypes, including M1s, M2 cells and small soma, low Opn4 expression cells (presumptive M5/M6 ipRGCs), and *Cdh3-GFP* cells (M6-type enriched). M2 and M3 types were combined during the process of coexpression analysis (designated M2/M3). Error bars represent SEM.

The T-box transcription factor Tbx20 was suggested from our gene expression analysis to be differentially expressed in ipRGCs. We selected it for further analysis in part for its potential role in maintaining the adult identity of ipRGCs. Tbx20 colocalization analysis with ipRGC subtypes was characterized as with our previous studies, but with the exception that M3 were combined with M2 types during ipRGC classification (annotated M2/M3) instead of with M1 types as in our other colabeling studies. Immunofluorescence colocalization analysis of Tbx20 and Opn4 expression confirmed its high expression in a subset of ipRGCs (Fig. 12). Tbx20 was expressed in most M1 cells (82.6 ± 1.8%, *n* = 514 across 4 retinas), but only in a minority of M2/3 cells (30.2 ± 6.5%, *n* = 1305) and low Opn4-IF cells (12.4 ± 3.4%, *n* = 603). One-half of the displaced M1 (dM1) cells expressed Tbx20 (46.0 ± 7.1%, *n* = 153). Strikingly, however, Tbx20 was not expressed at all in M4 cells (0%, *n* = 283).

**Figure 13.**
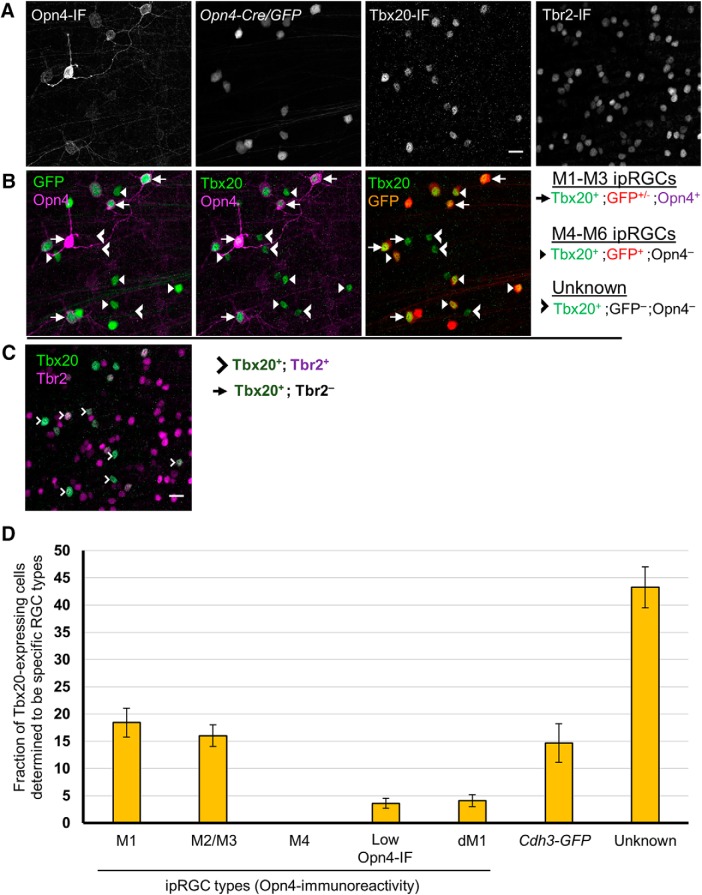
Coexpression study of Tbx20 with *Opn4-Cre/GFP* and Tbr2, including distribution of Tbx20-expression across ipRGC subtypes. ***A***–***C***, Quadruple immunofluorescence of Opn4, Tbx20, Opn4-Cre/GFP, and Tbr2. Scale bar, 20 μm. ***A***, Gray scale of Opn4, Opn4-Cre/GFP, and Tbx20 immunofluorescence. ***B***, Coexpression study of Tbx20 (green) in the context of Opn4 (magenta) and *Opn4-Cre/GFP* (red) labeling. GFP cells that are Opn4-immunonegative are inferred M4-M6 types. ***C***, Coexpression analysis of Tbr2 (magenta) with Tbx20 (green). ***D***, Distribution of Tbx20 expressing cells that belong to specific RGC types, to the extent that could be determined, including Opn4-immunoreactive ipRGC subtypes and RGCs labeled by the *Cdh3-GFP* transgenic reporter. Unaccounted Tbx20-expressing cells are designated as “unknown” RGC types. Error bars represent SEM.

Many Tbx20 cells were not detectably immunopositive for Opn4. Only 41% of Tbx20-immunopositive were also Opn4-immunoreactive (18.5 ± 2.6% were M1 cells; 16.0 ± 2.0% were M2/3 cells; and only 3.6 ± 0.9% were low Opn4-IF cells; *n* = 2184 across 4 retinas; Fig. 13). The remaining Tbx20-immunopositive cells were RGCs, as confirmed by Rbpms-immunoreactivity (data not shown). Additionally, Tbx20-immunopositive RGCs that were also Opn4-immunonegative were topographically enriched in the ventral retina, with most Tbx20-positive cells in the dorsal retina being accounted for by Opn4-immunoreactivity. These results demonstrate that Tbx20 is expressed in a diverse set of RGCs, including ipRGC subpopulations.

### Tbx20 expression in M5–M6 ipRGCs

To investigate whether some or all of the Tbx20-immunopositive RGCs that were Opn4-immunonegative might be ipRGCs of the M5 and M6 subtypes that exhibit weak Opn4 immunostaining, we examined the colocalization of Tbx20-immunopositive cells, Opn4-immunopositive cells, and all GFP-labeled cells in the *Opn4-Cre;Z/EG* mouse reporter, which among other ipRGCs, labels M5 and M6 cells. We observed examples of Tbx20-immunopositive cells that were GFP-positive (M1–M6 ipRGCs), but not Opn4-immunopositive M4-M6, and relatively small (not M4), suggesting that Tbx20 may be expressed in at least a subset of M5 or M6 ipRGCs (Fig. 13*A*,*B*).

To test the implication that many Tbx20 cells were M5 or M6, we turned to Cdh3-GFP mice. Most GFP+ RGCs in this mouse line are M6 cells and the remainder is M5 cells (Quattrochi et al., 2019). We tested Tbx20 immunoreactivity in the context of Opn4 immunofluorescence and *Cdh3-GFP* labeling (Fig. 12*B*,*C*). For the purpose of this study, we focused on GFP cells in the GCL that are Opn4-immunonegative (to distinguish from Opn4-immunopositive M2 types). We found that at 3 weeks after birth, most *Cdh3-GFP* cells express the Tbx20 protein (82.1 ± 4.3%, *n* = 439 across 4 retinas; Fig. 12*C*). Many, but not all, of the Opn4-immunonegative Tbx20-positive cells were GFP^+^ (27%, *n* = 1277 Tbx20^+^;Opn4^-^ cells; 4 retinas). The dorsal-ventral gradient of Tbx20-positive cells that are Opn4-immunonegative was broadly similar to the retinal labeling of the *Cdh3-GFP* reporter. A large portion of Tbx20-immunopositive cells remained unclassified (43.3 ± 3.8%, *n* = 2184; Fig. 13*D*).

Further, we determined whether Tbx20 expression correlates with the related T-box transcription factor Tbr2, a gene previously described to be enriched in adult ipRGCs (Mao et al., 2014; Sweeney et al., 2014). All Tbx20-expressing cells were strongly Tbr2-immunopositive (*n* = 328; 4 regions distributed across a single adult *Opn4-Cre/GFP* retina; Fig. 13*C*). Therefore, whereas Tbr2 is expressed in a broad range of types that includes the entire ipRGC family, Tbx20 expression is confined to a diverse subset of ipRGC subtypes.

### Molecular diversity of Rasgrp1 and Tbx20 expression in ipRGCs

**Figure 14.**
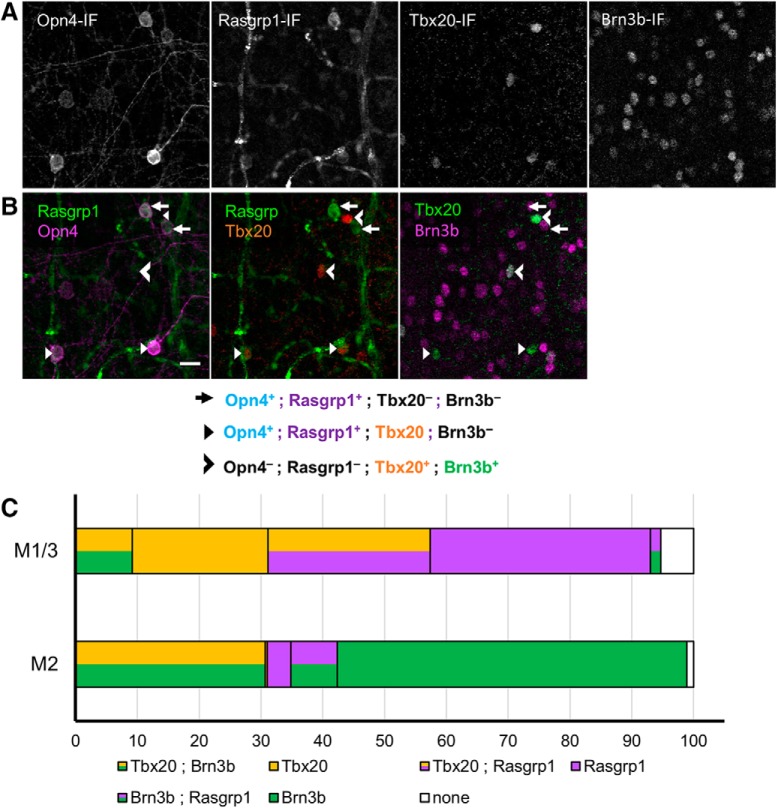
Complex pattern of Rasgrp1-Tbx20-Brn3b coexpression suggests further diversity in ipRGC family. ***A***, Quadruple immunofluorescence study of Tbx20, Brn3b, Opn4, and Rasgrp1 (gray scale). ***B***, Rasgrp1 and Opn4 (left) were initially quantified for ipRGC subtype expression before comparison with Tbx20 (middle) and Brn3b (right) expression. Rasgrp1, Brn3b, and Tbx20 expression are partially overlapping. ***C***, Integrated coexpression patterns of Brn3b, Rasgrp1, and Tbx20 with M1 and M2 ipRGC subtypes. The M1 group includes displaced M1 and M3 types.

Our expression studies revealed that Rasgrp1 and Tbx20 have a strikingly similar pattern of expression among ipRGC subtypes. Both genes were expressed in the majority of M1 cells, a minority of M2 cells, and a small population of low Opn4-IF cells, but not in M4 cells (Figs. 12 and 14). To directly test for coexpression, we compared and contrasted the expression patterns of Tbx20- and Rasgrp1-immunoreactivity in the context of the M1–M4 subtypes revealed by Opn4-immunoreactivity (Fig. 14). Rasgrp1 coexpression with Tbx20 was only observed in a fraction of M1/3 cells (26.0 ± 1.8%; *n* = 241 across 2 retinas, 2 mice; Fig. 14). Further, M1 cells expressing either Rasgrp1 or Tbx20 alone accounted for approximately similar fractions of M1 cells (37.3 ± 4.0% and 31.1 ± 3.5%, respectively). Only a small fraction of M1 cells were immunonegative for both Rasgrp1 and Tbx20 (5.3 ± 0.7%). In contrast, one-half of M2 cells lacked Rasgrp1 and Tbx20 immunoreactivity (57.7 ± 3.3%; *n* = 388 across 2 retinas, 2 mice). Approximately one- third of M2 cells expressed Tbx20 (31.0 ± 0.7%), whereas only 11.3 ± 5.1% expressed Rasgrp1. We did not observe any example of an M2 cell expressing both Rasgrp1 and Tbx20.

### Molecular diversity of SCN-projecting ipRGCs

We further examined the Rasgrp1- and Tbx20-expressing ipRGC subtypes to seek intersectional expression patterns that would divide ipRGCs by their downstream visual pathways. Earlier studies showed that M1 cells could be subdivided based on their level of expression of Brn3b (Chen et al., 2011; Jain et al., 2012). We used quadruple immunolabeling to simultaneously test Brn3b expression with Rasgrp1- and Tbx20-immunoreactivity in the context of Opn4-immunolabeled ipRGCs (25 regions, 3 wild-type retinas; Fig. 14*A*,*B*). We determined that a minority of M1/3 cells express Brn3b (7.9 ± 6.0%, *n* = 241), which is similar to previous studies (Jain et al., 2012). The Brn3b^+^ M1/3 cells expressed either Tbx20 or Rasgrp1 (91.0 and 9.0 ± 10.1%, respectively; *n* = 30; Fig. 14*C*).

Further, we determined that most M2 cells expressed Brn3b (90.8 ± 6.9, *n* = 168). In contrast to M1/M3 ipRGCs, the majority of Brn3b^+^ M2 ipRGCs did not express either Rasgrp1 or Tbx20 (67.4.6 ± 13.8, *n* = 222). Most M2 cells expressing Tbx20 were also Brn3b-immunopositive (84.5 ± 25.1, *n* = 118). The small subset of M2 cells that express Rasgrp1 could be further divided by Brn3b presence or absence (5.0 ± 6.0% and 4.5 ± 1.4%, respectively; *n* = 168). Generally, we found no cells coexpressing all three genes (*n* = 729).

**Figure 15.**
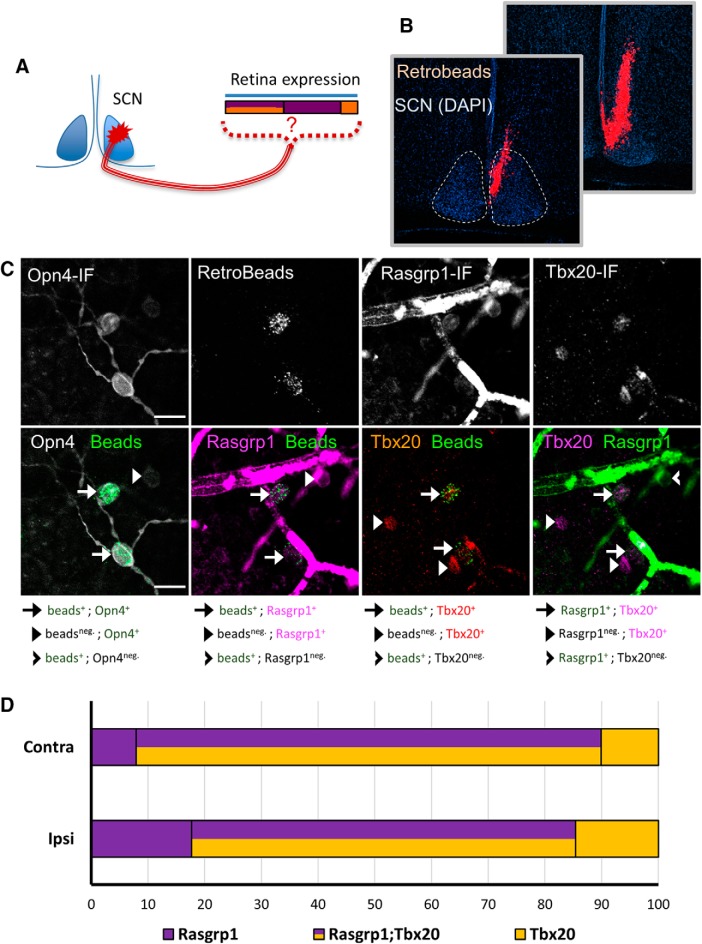
The ipRGCs projecting to the SCN have a molecularly diverse pattern of Rasgrp1 and Tbx20 expression. ***A***, Experimental design of fluorescent bead injection to SCN, followed by examination of Rasgrp1 and Tbx20 expression in retrograde labeled RGCs. ***B***, Neuro-anatomical study to verify that retrograde injection is within the SCN, but not the optic nerve. ***C***, Triple immunofluorescence of Opn4, Rasgrp1, and Tbx20 in combination with fluorescent Retrobeads. Retrobeads were mostly observed in Opn4-immunopositive RGCs (M1–M3 ipRGCs). Quantification of Rasgrp1 and Tbx20 in retrolabeled cells. ***D***, SCN-projecting ipRGCs in the ipsilateral and contralateral retina are molecularly diverse for Tbx20 and Rasgrp1 expression.

Although Brn3b has been used as a proxy for distinguishing M1 ipRGC subpopulations targeting distinct brain regions, many M1 cells have transient Brn3b expression in development that is downregulated by adulthood (Chen et al., 2011). Therefore, we directly correlated gene expression of Rasgrp1 and Tbx20 in the retina with retrograde labeling from the SCN (Fig. 15*A*). We injected rhodamine-conjugated retrobeads in the SCN, followed by immunofluorescence labeling for Opn4, Rasgrp1, and Tbx20 (Fig. 15*A*–*C*). All injection sites clearly involved the SCN, as revealed by DAPI labeling, but did not spread to the optic chiasm or tract (Fig. 15*B*). Quantitative coexpression analysis (18 confocal images collected across the contralateral and ipsilateral retinas) revealed that nearly all retrolabeled cells were Opn4-immunopositive (95.2%, 248 retrolabeled cells). Most retrolabeled Opn4-immunoreactive cells expressed both Rasgrp1 and Tbx20 (79 ± 5%, across 18 sections, 235 cells), but equal minorities expressed either Rasgrp1 (10 ± 3%) or Tbx20 (10 ± 4%; Fig. 15*C*). This expression pattern was consistent across the ipsilateral and contralateral retina (Fig. 15*D*), as suggested by a bilateral input to the SCN (Hattar et al., 2006; Fernandez et al., 2016). Therefore, we show that SCN-projecting ipRGCs have a complex pattern of Rasgrp1 and Tbx20 gene expression. Together, these results provide evidence for previously unrecognized molecular diversity in adult ipRGCs.

## Discussion

Prior efforts to assess the distinctive genetic composition of ipRGCs have been complicated by their rarity among diverse retinal cell types and the inherent difficulties of maintaining viability of dissociated mature neurons (Lobo et al., 2006). Our approach was first to isolate RGCs by immunoaffinity, then to further purify ipRGCs from these based on genetic labeling and FACS, and to finally to compare the transcriptional profiles of the purified ipRGCs to those of the residual cell pool, consisting mainly of conventional RGCs. The relative purity of our ipRGC sample is supported by enrichment for transcripts of genes known to be differentially expressed in ipRGCs and the low levels of transcripts selectively expressed in potentially contaminating populations, including the abundant rod photoreceptors. Our isolation method and differential expression analysis allowed us to identify >75 DEGs in ipRGCs relative to conventional RGCs.

### Genes differentially expressed in adult ipRGCs

There is limited knowledge of specific gene expression in ipRGCs generally and within particular ipRGC subtypes, especially non-M1 ipRGCs. Many diverse genes appeared more highly expressed in ipRGCs than in conventional RGCs. We confirmed differential protein expression in ipRGCs immunohistochemically for two of these genes: Tbx20, a transcription factor implicated in visual development; and Rasgrp1, a G-protein exchange factor that may interact with the melanopsin phototransduction cascade. However, only a subset of ipRGCs appeared to express detectable levels of these proteins, and such variable expression was apparent even among ipRGCs of the same subtype. Some ipRGCs expressed both proteins, but many did not. This diversity even extended to the M1 cells projecting to the SCN, which had been thought to share the distinctive molecular feature of little or no expression of the transcription factor Brn3b. These novel markers of molecularly distinctive ipRGC varieties open the way for cell-type-specific manipulations through intersectional strategies.

### Which type(s) of adult ipRGCs express Rasgrp1?

Rasgrp1 expression has previously been detected in the hippocampus, striatum and olfactory regions of the brain (Ebinu et al., 1998; Toki et al., 2001), but our study appears to be the first to explore Rasgrp1 expression in the eye. Rasgrp1-like immunoreactivity marked a diverse subpopulation of ipRGC subtypes, including the M1-M3 ipRGC subtypes but not the M4-type. Either M5 or M6 ipRGCs, or both, also appear likely to express Rasgrp1, because some Rasgrp1-immunoreactive cells had weak Opn4-immunoreactivity without the characteristic dendritic labeling of M1–M3 ipRGCs and with somas too small to be M4 cells (Ecker et al., 2010; Stabio et al., 2018; Quattrochi et al., 2019).

We find Rasgrp1 to be expressed not only in SCN-projecting M1 ipRGCs, but also in other ipRGC subtypes, especially M2 cells and apparently M5 and/or M6 cells. Collectively, these types project to various non-image-forming visual centers, including the OPN, intergeniculate leaflet, and dorsal lateral geniculate nucleus (Stabio et al., 2018; Quattrochi et al., 2019).

The function of Rasgrp1 in ipRGCs is unknown, but it could interact with the melanopsin phototransduction cascade. The direct photoresponse of ipRGCs appears to increase levels of both DAG and calcium. Both of these signaling molecules bind to and activate Rasgrp1, and trigger its translocation to the Golgi apparatus (Bivona et al., 2003; Graham et al., 2008; Zhang et al., 2010). However, ipRGC phototransduction Rasgrp1 signaling does not appear to be essential for ipRGC phototransduction because more than a quarter of M1 ipRGCs and the great majority of M2 cells are immunonegative for Rasgrp1. Even in ipRGCs, Rasgrp1 may be activated by DAG and calcium signals unrelated to Opn4 phototransduction, and such signals are presumably also responsible for modulating Rasgrp1 in cells (such as certain amacrine cells), which express Rasgrp1 but not melanopsin.

Rasgrp1 has the potential to affect any number of neuronal signaling pathways. Ras signaling pathways are enormously complex and the cross talk between pathways makes it even harder to identify specific effects. One basic mechanism for specificity in Ras signaling is the distinct subcellular targeting of downstream components of the signaling pathway. In lymphocytes, localized Ras signaling of Rasgrp1 occurs preferentially on the Golgi apparatus, which is a rare form of compartmentalized Ras signaling (Bivona et al., 2003; Zhang et al., 2010). The Golgi apparatus in neurons provides the posttranslational protein modifications required for organizing protein and organelle trafficking throughout the cell. Rasgrp1 could play a crucial role in orchestrating a specific set of post-translational modifications at the Golgi.

### Tbx20 is expressed in a diverse set of ipRGC subtypes

The T-box transcription factor Tbx20 exhibited enriched expression relative to conventional RGCs in postnatal and adult retinas. Double immunolabeling revealed that many ganglion cells that expressed this protein also expressed melanopsin. Similar to Rasgrp1, Tbx20 was determined to be expressed in most M1 ipRGCs (83%), a substantial minority of M2 cells (30%) and an additional population of RGCs whose identity was not immediately obvious. We decided to then compare Tbx20 against other known molecular patterns in ipRGCs. Whereas most RGCs follow a Brn3b-dependent developmental program, the M1 ipRGCs that project to the SCN do not express Brn3b, whereas OPN-projecting M1 ipRGCs express Brn3b. We found that Brn3b-expressing M1 cells are also Tbx20-immunopositive. The Brn3b-negative M1 cells are split between cells that express Tbx20 and those that do not. This finding suggests that ipRGCs are more molecularly diverse than originally anticipated: Tbx20 is expressed in ipRGCs with differing brain targets, Tbx20 is expressed across multiple morphologically defined subtypes, and Tbx20 is not expressed in all of any one type. The exploration of Tbx20 coexpression with Rasgrp1 revealed a complex coexpression pattern among M1-M3 ipRGCs.

Tbx20 has well-established roles in embryonic development and is continuously required in mature neurons and other cell types to maintain their identities and functional properties during adulthood (Naiche et al., 2005). Tbx20 functions as a repressor in early embryonic ocular development (Carson et al., 2000; Pocock et al., 2008) and is required for the expansion of the small pool of precursor cells in the optic vesicle (Carson et al., 2004). However, little is known about the function of Tbx20 in the adult retina.

Tbx20 can function as a transcriptional activator in parallel with its repressor activity, with these two roles impinging on distinct biological processes (Sakabe et al., 2012). In addition to its key developmental roles, Tbx20 appears vital for maintaining the structure and function of cardiac muscle cells in the adult mouse heart (Stennard et al., 2003; Shen et al., 2011). In adult cardiomyocytes, Tbx20 is responsible for directly activating genes critical for normal adult cardiac function such as those required for ion transport and heart contraction (Shen et al., 2011; Sakabe et al., 2012). In contrast, genes directly repressed by Tbx20 have known roles in non-heart developmental programs, cell cycle, proliferation, and immune response (Sakabe et al., 2012). The transcriptional effects of Tbx20 shift during cardiac development, from early mediation of proliferation of cardiac progenitors, to implementation of an anti-proliferative program in the adult heart (Cai et al., 2005; Takeuchi et al., 2005). Therefore, Tbx20 cooperates with distinct cohorts of transcription factors to either promote or repress distinct molecular programs in a context-dependent manner (Sakabe et al., 2012). Similarly, binary cell fate specification in the retina is driven by complex genetic programs that require the simultaneous activation and repression of genes by transcription factors. Tbx20 may prove to have a similar reversal in its transcriptional activity in the retina when transitioning from broad embryonic development program to regulating adult neuron identity of a subset of ipRGCs. Further, Tbr2 is another Tbox family member that is known to have a critical role in the development of RGCs (Mao et al., 2008), which later becomes essential to a restricted set of ipRGCs that participate in non-image forming visual circuits (Mao et al., 2014; Sweeney et al., 2014). Our studies determined that Tbx20 and Tbr2 are coexpressed in adult ipRGCs. They may work cooperatively to specify ipRGC subtype identity by regulating cell-specific transcriptional programs and repressing alternate fates.

### Characterization of ipRGC subtypes

Retinal cell types are generally classified using a combination of morphology, gene expression, mosaic organization, light responses and synaptic connectivity (Sanes and Masland, 2015). By these criteria, intrinsically photosensitive RGCs comprise at least six distinct cell types. Though all express melanopsin, they differ from one another in the strength of the intrinsic response, their morphology, pattern of light responses, and projections to the brain. However, the further subdivision may be in order. The M1 type appears subdivisible into at least two subtypes, one expressing the transcription factor Brn3b and innervating the OPN and geniculate complex, while the other lacks Brn3b expression and innervates the SCN (Chen et al., 2011). Our study shows further diversity in the M1 and M2 types based on the expression of Rasgrp1 and Tbx20. For example, we find molecular diversity among in the SCN-projecting ipRGC subtypes. It is unclear to us whether this should be used to propose a further formal subdivision of M1 and M2 cells. For example, the expression of these proteins could fluctuate over time in individual cells and be uncorrelated across cells of the same type.

### Comparison with other ipRGC gene expression profiles

Siegert et al. (2012) surveyed gene expression in many different sets of mouse retinal neurons, using specific mouse reporters strains (including the *Opn4-Cre* reporter system for ipRGCs), FACS isolation of labeled cells, and microarray analysis. Many of the genes they found strongly expressed in ipRGCs were also among the genes we found differentially expressed in ipRGCs. However, dozens of additional genes differentially expressed in ipRGCs emerged from our analysis that were not detected in theirs (Siegert et al., 2012). Discrepancies between their findings and ours may stem from technical factors such as differing degrees of contamination of the starting material with rod photoreceptor transcripts, the use of internal control cell populations for relative gene expression comparison in our study but not theirs, and differences between microarray and RNA-sequencing methodologies.

Another study used single-cell transcriptomic analysis of the mouse retina and were able to identify ipRGCs from their cell suspensions (Macosko et al., 2015). Macosko et al., 2015 distinguished the main broad class of RGCs, but they required *posthoc* supervised analysis to distinguish a limited number of genes attributed to Opn4-positive cell clusters. They identified nine genes with a twofold increase in expression compared with Opn4-negative cells. Three genes (Tbr2, Igf1, and Tbx20) were also found to be enriched in our ipRGC samples. In contrast, Tbx20 did not reach above threshold for Siegert et al. (2012), but it is among the highest expressing ipRGC-enriched genes in our study. Our study complements a recently published single-cell RNA-seq study of RGCs from pre-eye opening age (P5) mice (Rheaume et al., 2018). Although ipRGCs were not a major focus, Rheaume et al. (2018) recognized ipRGCs by clustering and considering established markers such as Opn4 and Eomes/Tbr2 in clusters 6, 25, 26, 33, and 37. They identified further molecular diversity that maps well onto known ipRGC subtypes and those described in our current study. Rheaume et al. (2018), determined that the ipRGCs clusters 25 and 37 have major expression of Tbx20, with minor expression in cluster 6. Although not explicitly mentioned, their dataset also includes restricted Rasgrp1 expression in clusters 6 and 25. Considering our gene expression datasets and coexpression studies of Rasgrp1 and Tbx20 in subsets of M1 and M2 cells, we suggest that clusters 6 and 25 constitute these types. Further, we infer that clusters 26 and 37 represent M5 and M6 cells, respectively, considering the following information: (1) Cdh3 expressed in clusters 6, 26, and 37. The *Cdh3-GFP* reporter preferentially labels bistratified M6 ipRGCs, but also labels some monostratified M2 and M5 ipRGCs (Quattrochi et al., 2019); (2) *Cdh3-GFP* labeled RGCs express Cdh6 (Osterhout et al., 2011), which is expressed in clusters 26 and 37; and (3) our study determined that Tbx20 was highly expressed in *Cdh3-GFP* reporter, suggesting enriched expression in M6 ipRGCs. Important differences remain between the experimental parameters of these two studies. Droplet-based scRNA-seq technologies used by Rheaume et al. (2018) provides hierarchies of molecular types and classes, but the sequencing is relatively shallow and our complementary approach of deep sequencing purified populations of ipRGCs generates more complex cDNA libraries with increased sensitivity of detected gene enrichment. In addition to postnatal age group similar to Rheaume et al. (2018), we expanded our gene expression profiles to adult ipRGCs, which required substantial optimizations of our dissociation and FACS acquisition process to obtain viable samples of the relatively fragile adult neurons.

### Further considerations

Our identification of DEGs in ipRGCs should be treated as hypothesis for genes that are functionally relevant for ipRGC function. Although our selection criteria dramatically decreased the potential heterogeneity obscuring differential gene expression in ipRGC, the transgenic reporters used in our studies are known to label multiple morphologically and physiologically distinct ipRGC types. For example, genes determined to be differentially expressed using the *Opn4-GFP* reporter can, at best, be inferred to have restricted expression within the M1–M3 ipRGC types. However, it would be impossible to know whether this gene is expressed in all M1–M3 types or a subset. Further, we cannot assume that DEGs in our studies are uniquely expressed in ipRGCs and absent in non-ipRGCs. Our studies are based on relative abundance of ipRGCs compared with cRGC populations. As exemplified by Tbx20, we cannot rule out scenarios that DEGs are enriched in ipRGCs, but are also expressed in additional subsets of other RGCs.

The study of transcriptomes and differential gene expression has certainly proven important for revealing otherwise unknown molecular underpinnings to specialized cell function. However, the transcription of genomic DNA to mRNA is only one of many intermediate steps to the synthesis of functional proteins. Translational control, post-translational modifications, and subcellular localizations are examples of ways that the level and function of proteins can be decoupled from the relative abundance of mRNA expression. Therefore, additional follow-up localization studies using *in situ* hybridization or immunofluorescence will be required to test the validity and cell-type-specific expression of our proposed DEGs.

## Conclusion

In conclusion, our results demonstrate a method to purify ipRGCs and identify an extensive list of >75 genes that are differentially expressed in adult ipRGCs compared with generic RGCs. These genes will be useful for the identification of marker genes for ipRGC subtypes, comparison of gene expression across types, understanding the intracellular gene networks underlying ipRGC phenotypes, and the testing for conservation of ipRGC molecular programs across mammalian species.

## Synthesis

Reviewing Editor: Tatyana Sharpee, The Salk Institute for Biological Studies Decisions are customarily a result of the Reviewing Editor and the peer reviewers coming together and discussing their recommendations until a consensus is reached. When revisions are invited, a fact-based synthesis statement explaining their decision and outlining what is needed to prepare a revision will be listed below. Note: If this manuscript was transferred from JNeurosci and a decision was made to accept the manuscript without peer review, a brief statement to this effect will instead be what is listed below.

Please revise the manuscript to address comments from the reviewers and provide a point-by-point description of the changes made. I would like to send the manuscript to the original reviewers.
